# The genetics and physiology of seed dormancy, a crucial trait in common bean domestication

**DOI:** 10.1186/s12870-021-02837-6

**Published:** 2021-01-22

**Authors:** Ali Soltani, Katelynn A. Walter, Andrew T. Wiersma, James P. Santiago, Michelle Quiqley, Daniel Chitwood, Timothy G. Porch, Phillip Miklas, Phillip E. McClean, Juan M. Osorno, David B. Lowry

**Affiliations:** 1 Plant Resilience Institute, Michigan State University, East Lansing, MI USA; 2 Department of Plant Biology, Michigan State University, East Lansing, MI USA; 3 Department of Plant, Soil and Microbial Sciences, Michigan State University, East Lansing, MI USA; 4 Department of Horticulture, Michigan State University, East Lansing, MI USA; 5 Department of Computational Mathematics, Science and Engineering, Michigan State University, East Lansing, MI USA; 6 USDA-ARS, Tropical Agriculture Research Station, Mayaguez, PR USA; 7 USDA-ARS, Grain Legume Genetics Physiology Research Unit, Prosser, WA USA; 8 Department of Plant Sciences, North Dakota State University, Fargo, ND USA

**Keywords:** Common bean, Domestication, Dormancy, Germination, Imbibition, Pectin

## Abstract

**Background:**

Physical seed dormancy is an important trait in legume domestication. Although seed dormancy is beneficial in wild ecosystems, it is generally considered to be an undesirable trait in crops due to reduction in yield and / or quality. The physiological mechanism and underlying genetic factor(s) of seed dormancy is largely unknown in several legume species. Here we employed an integrative approach to understand the mechanisms controlling physical seed dormancy in common bean (*Phaseolus vulgaris* L.).

**Results:**

Using an innovative CT scan imaging system, we were able to track water movements inside the seed coat. We found that water uptake initiates from the bean seed lens. Using a scanning electron microscopy (SEM) we further identified several micro-cracks on the lens surface of non-dormant bean genotypes. Bulked segregant analysis (BSA) was conducted on a bi-parental RIL (recombinant inbred line) population, segregating for seed dormancy. This analysis revealed that the seed water uptake is associated with a single major QTL on Pv03. The QTL region was fine-mapped to a 118 Kb interval possessing 11 genes. Coding sequence analysis of candidate genes revealed a 5-bp insertion in an ortholog of *pectin acetylesterase 8* that causes a frame shift, loss-of-function mutation in non-dormant genotype. Gene expression analysis of the candidate genes in the seed coat of contrasting genotypes indicated 21-fold lower expression of *pectin acetylesterase 8* in non-dormant genotype. An analysis of mutational polymorphism was conducted among wild and domesticated beans. Although all the wild beans possessed the functional allele of *pectin acetylesterase 8*, the majority (77%) of domesticated beans had the non-functional allele suggesting that this variant was under strong selection pressure through domestication.

**Conclusions:**

In this study, we identified the physiological mechanism of physical seed dormancy and have identified a candidate allele causing variation in this trait. Our findings suggest that a 5-bp insertion in an ortholog of *pectin acetylesterase 8* is likely a major causative mutation underlying the loss of seed dormancy during domestication. Although the results of current study provide strong evidences for the role of *pectin acetylesterase 8* in seed dormancy, further confirmations seem necessary by employing transgenic approaches.

**Supplementary Information:**

The online version contains supplementary material available at 10.1186/s12870-021-02837-6.

## Background

The domestication of plants was a crucial step in the emergence of agriculture and the rise of human civilization [1]. The process of domestication resulted in plants adapted to human-managed agricultural environments. These adaptations occurred through a process of selection within wild species for genotypes with a suite of traits that provided a selective advantage in these new habitats. In many cases, analogous selection practices resulted in phenotypic convergence of domestication traits across species [2]. Common domestication traits in the majority of crops include loss of seed shattering, seed dormancy, and more synchronous germination [2–6]. Although domestication traits are favorable in agricultural ecosystems, they impose a trade-off for fitness in natural habitats [1, 7, 8].

A reduction in seed dormancy is an important process that is commonly associated with domestication [2, 3, 9]. In a natural environment, the timing at which seeds break dormancy is crucial, as germination at the wrong time can result in reduced survival and fitness [9, 10]. Besides synchronization with the environment, seed dormancy is involved in seed dispersal and in the reduction of resource conflicts between mother and offspring [11]. In this scenario seeds with longer dormancy can be dispersed more widely in time and space and consequently reduce the resource conflicts between mother and offspring and/or among offspring.

Despite being advantageous in natural environments, extended seed dormancy is not a desirable trait for crops [2, 3, 9]. In cultivated legumes, seed dormancy reduces the rate of germination, which results in uneven germination and consequently, decreased yields [12, 13]. Further, physical seed dormancy (see below) adversely affects water uptake by the seed, which is important in food processing of legumes [9]. While a high level of dormancy is not desirable, low levels of seed dormancy can be problematic for crops where germination occurs before seed harvest, a process known as vivipary in cereals [14] and legumes [15]. Vivipary is a major issue in environments that are favorable for germination. Thus, achieving the optimal balance between rapid germination and seed dormancy is critical for maximum potential agricultural yield and grain processing [9].

Several mechanisms promote seed dormancy [16]. Physiological dormancy, where hormonal interactions, particularly between abscisic acid (ABA) and gibberellins (GA), plays an important role in inducing and maintaining dormancy and is thought to be the most common mechanism of seed dormancy [16]. Physical characteristics of the seed, especially the seed coat, can also promote longer dormancy by preventing uptake of water. This physical dormancy is the most phylogenetically restricted and is believed to be an adaptation mechanism for habitat specialization [16].

Physical dormancy appears to be the most prevalent cause of dormancy in legumes, while physiological dormancy is a factor in some legume and non-legume species [17].

The underlying mechanism for physical seed coat impermeability varies between legume species. While the underlying genetic mechanism is largely unknown, changes in the level of dormancy is controlled by a few loci in some legume species [4, 18–22]. From a physiological perspective, impermeable seeds often have a hard, pectinaceous outer layer of palisade cells [23] or a higher lignin content in the seed coat [24]. Seed impermeability can also be associated with fatty acid composition of the cutin layer of the seed coat [25]. Seed coat in legumes (testa) is originated from mother tissues and consists of two ovular integuments (bitegmic ovule, [26]). The inner layer largely disappears during seed development [27]. However, the outer layer develops into the seed coat and becomes impermeable to water in dormant seeds. Physical seed dormancy can be decreased through the development of micro-cracks on the seed coat [28].

Common bean (*Phaseolus vulgaris* L.) is an important legume species for direct human consumption. Prior to domestication, *P. vulgaris* migrated from its center of origin in Mexico to Central and South America. *P. vulgaris* was then domesticated independently in Mesoamerica and the Andes [29, 30]. The successful adoption of common beans by farmers in different continents with diverse climates highlights its vast genetic diversity and broad adaptation. Although early Andean farmers (2500 years ago until 600 years ago) maintained genetic diversity, modern breeding practices resulted in a great loss of genetic diversity in cultivated Andean beans [31].

In modern common bean production, beans with high, uniform rates of water uptake are considered desirable [32]. High water uptake reduces cooking time. Furthermore, the water uptake rate influences the swelling capacity that determines the number of cans that can be produced from a unit of dry beans [33].

In previous studies we found that genotypes with the lowest seed imbibition rates had the highest rates of survival under flooding conditions [34, 35]. This finding suggests that there is a trade-off between the benefits of rapid seed water uptake and susceptibility to flooding. Furthermore, seed dormancy likely benefited natural populations of *P. vulgaris* prior to domestication by preventing germination during unfavorable environmental conditions, as it does in many other plant species [16, 36]. Despite the benefits of rapid water uptake for processing and cooking beans, some domesticated bean varieties still retain a high level of seed dormancy [34, 35, 37, 38] making them more tolerant to flooding.

To investigate the genetic and physiological basis of divergence in the rate of seed water uptake among domesticated bean varieties we focused our study on two representative common bean genotypes that differ greatly in their rate of seed water uptake [35], PR9920–171 [39] and TARS-HT1 [40]. PR9920–171 was the most tolerant genotype to flooding at the germination stage among a panel of Andean genotypes [35]. TARS-HT1 is a dark red kidney, and PR9920–171 is a mottled light red kidney both were developed as improved germplasm with substantial tolerance to heat stress. Both PR9920–171 and TARS-HT1 were derived from crosses with Indeterminate Jamaica Red (hereafter IJR, PI 163122), which is a slow-imbibing genotype with a high level of flooding and heat tolerance.

In this study, we compared the seed traits of PR9920–171 and TARS-HT1 and conducted genetic analysis of crosses between these two lines to address four major questions: *i*) What is the physiological mechanism underlying seed dormancy in common bean? *ii*) How does increased dormancy correspond with higher tolerance to flooding during germination? *iii*) What is the genetic basis of seed dormancy? and *iv*) Are the same genetic mechanisms underlying the divergence in seed dormancy within domesticated bean also responsible for its divergence from wild populations of *P. vulgaris*?

## Results

### Seed germination and imbibition

**Fig. 1:**
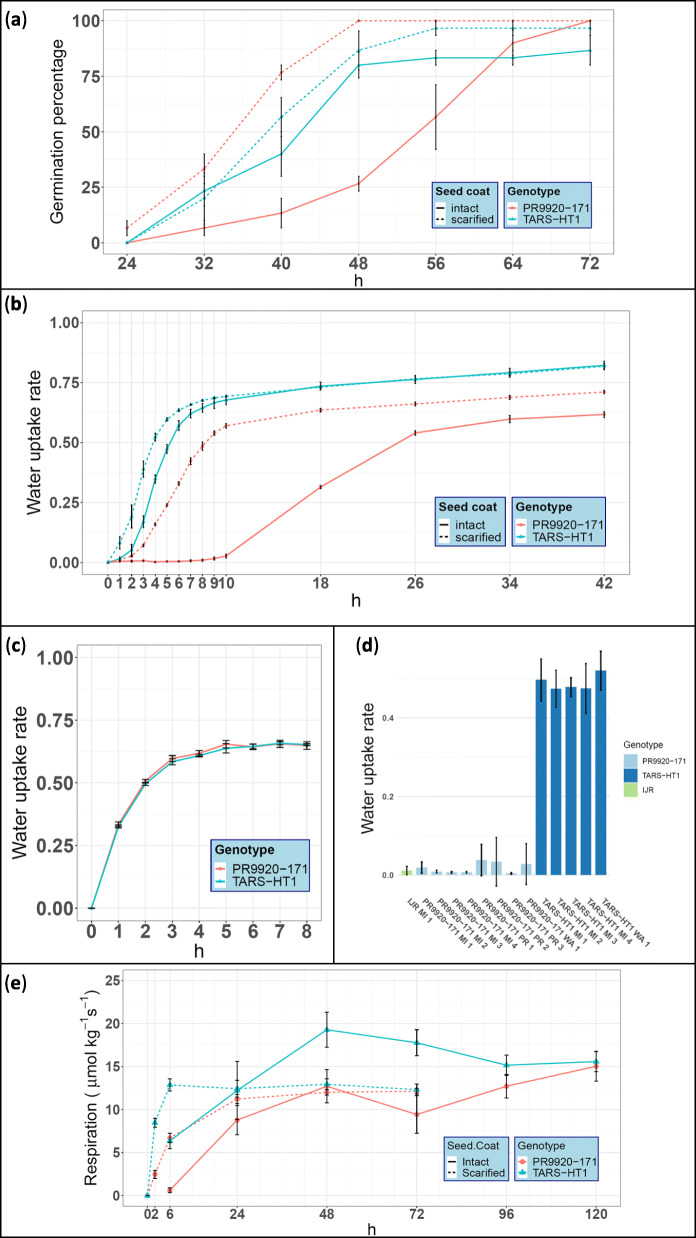
The difference between TARS-HT1 and PR9920–171 for physical dormancy. **a** The germination assay performed on both genotypes with intact and scarified seed coat. This experiment revealed that PR9920–171 possessed strong physical dormancy. **b** The imbibition assays performed on both beans with intact and scarified seed coat. TARS-HT1 beans imbibed water at a faster pace compared to PR9920–171 beans. Seed coat scarification significantly enhanced imbibition for both genotypes, particularly PR9920–171 beans, indicating strong physical dormancy of this genotype. **c** Imbibition assay of cotyledons after removal of the seed coats. No significant differences were detected for imbibition rate of the cotyledons, indicating the seed coat is the main contributor of physical dormancy in PR9920–171. **d** Water uptake rate of PR9920–171 and TARS-HT1 beans grown at three field environments in MI, WA and PR in 2019. In all locations, the PR9920–171 had a significantly lower imbibition rate compared to TARS-HT1. IJR the parental lines of both genotypes showed a slow-imbibition phenotype similar to PR9920–171. The numbers in front of each location indicates the replication number. **e** Respiration measurement of seeds at different time-points after soaking for PR9920–171 and TARS-HT1 genotypes. The respiration rate in TARS-HT1 seeds followed a faster rate compared to PR9920–171 seeds. The intact seeds of TARS-HT1 reached their maximum respiration capacity at 48 h after soaking. In contrast, the respiration rate in PR9920–171 did not decrease in respiration even after 120 h of soaking. In panel **a**, **b**, **c**, and **e**, red and blue lines indicate PR9920–171 an TARS-HT1, respectively. Solid and dashed lines indicate the beans with intact and scarified seed coats, respectively

The seed germination assay revealed that PR9920–171 possessed a strong physical dormancy that can be restored by seed coat scarification (Fig. 1a). The most drastic difference for germination percentage was observed at 48 h after soaking. At this time point, although PR9920–171 seeds with intact seed coats showed 25% germination, scarification increased the germination of PR9920–171 to 100%. At the same time point, TARS-HT1 had ~ 77% germination.

TARS-HT1 seeds with intact seed coats start imbibing water within 2 hours of soaking (Fig. 1b). In contrast, the first sign of PR9920–171 seed imbibition was observed around 10 h after soaking was initiated. For both genotypes, we observed that seed coat scarification significantly increased the rate of imbibition compared to those with intact seed coats. This enhancement in imbibition rate was particularly evident for PR9920–171 seeds, which suggests that the seed coat imposes a greater obstacle for water penetration into the seeds in this genotype. At 8 and 26 h after soaking, the imbibition rate reached a plateau for TARS-HT1 and PR9920–171, respectively, but at significantly different levels. The further increase in seed weight was associated with radicle elongation rather than imbibition.

To test the hypothesis that cotyledons differ in their water uptake rate between the genotypes, the imbibition assay was performed on the cotyledons without a seed coat. For both genotypes, the maximum imbibition (water uptake) of the cotyledons was reached within 4 h of soaking (Fig. 1c). No significant differences were detected between the imbibition rate of cotyledons without seed coats, further confirming that the seed coat is the sole contributor to the lower imbibition rate of PR9920–171.

Field evaluation in three field locations indicated the stability of seed dormancy in PR9920–171 (Fig. 1d). On average, the water imbibition in TARS-HT1 seeds was more than 5-fold greater than PR9920–171 seed imbibition over three locations. Furthermore, PR9920–171 imbibition was similar to IJR imbibition.

### Seed respiration

We observed that TARS-HT1 seeds with intact seed coats reached their highest respiration capacity at 48 h after the initiation of soaking (Fig. 1e) and decreased throughout the remainder of the 96 h assay. In contrast, the respiration rate of PR9920–171 intact seeds also increase through 48 h, but at a lower level, and it reached the same level as TARS-HT1 at the end of the assay (15.0 ± 1.7 μmol kg ^− 1^ s^− 1^ at 120 h). This indicates that PR9920–171 seeds took longer to reach their maximum respiration capacity under soaking conditions. Respiration for seeds with scarified seeds coats had a much faster trajectory for both genotypes when compared to seeds with intact seed coats. Scarified TARS-HT1 seeds reached their respiration capacity (12.9 ± 0.7 μmol kg ^− 1^ s^− 1^) within 6 h of soaking and remained at that plateau until 72 h. However, the respiration rate increase was slower for PR9920–171.

### The lens structure is the potential site of water entry into bean seeds

**Fig. 2:**
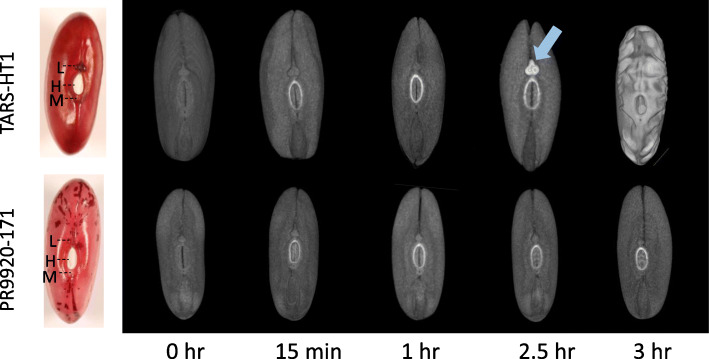
Tracking seed water uptake in PR9920–171 and TARS-HT1 using iodine contrast and CT-scan imaging. Within one hour of soaking in iodine solution, a bright signal around the hilum was detected in both genotypes. At 2.5 h, a change was detected on the lens (arrow) of TARS-HT1 seeds which indicates the site of water entry in the seed coat. After 3 h of soaking, the water penetrated the whole seed coat of TARS-HT1 while PR9920–171 seeds remained unimbibed. The seeds of both genotypes were represented on the left as a reference. Several structures are distinguishable in legume seeds. H = hilum, L = Lens and M = micropyle

After 30 min, a higher density region around the hilum was detected in CT-scanned seeds for both slow and fast-imbibing genotypes (Fig. 2). However, this initial water absorption was not accompanied by further water uptake. Around 2.5 h after soaking, the lenses of the fast-imbibing genotypes were distinguishable from other parts of the seed by higher water-iodine uptake (Fig. 2). However, the seeds in slow-imbibing genotypes did not imbibe water in that time-span. This indicates that imbibition in these common bean genotypes is associated with the initial water uptake through the lens structure. The lens or strophiole appears as two swelling structures in the common bean testa. We blocked the lens of both genotypes with nail polish and assessed their imbibition. The lens blockage did not seem to prevent imbibition in the TARS-HT1 seeds. However, in PR9920–171, lens blockage completely prevented seed imbibition (Figure S2). This result revealed that the lens is important for imbibition in genotypes with strong physical dormancy.

### Fast imbibing genotypes possess more micro-cracks on the surface of the seed lens

**Fig. 3:**
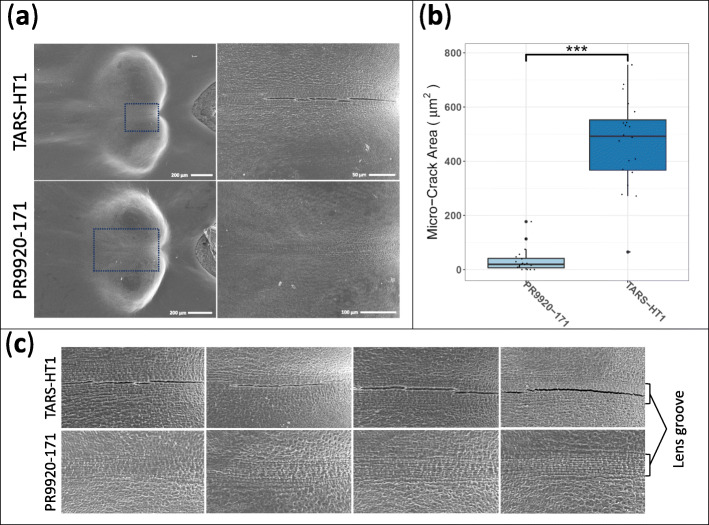
Scanning electron microscopy imaging of the lens structures of fast-imbibing (TARS-HT1) and slow-imbibing (PR9920–171) genotypes. **a** Large micro-cracks were detected on the lens of TARS-HT1. **b** The micro-cracks area was wider on the lens of TARS-HT1. **c** Different shapes of micro-cracks were detected alongside of the lens groove. The lens groove cells were better sealed in PR9920–171

SEM revealed that the fast-imbibing seeds possess several micro-cracks alongside the lens surface (Fig. 3a). We found that micro-crack areas (μm^2^) on the lens surface of TARS-HT1 seeds were about 14-fold greater (*P* < 0.001, *n* = 20) than in the slow-imbibing genotype (PR9920–171, Fig. 3b). These micro-cracks appeared alongside the lens groove of TARS-HT1 (Fig. 3c).

### A single major QTL on Pv03 (*P. vulgaris* chromosome 3) underlies variation in seed dormancy

We employed a bulked segregant analysis (BSA) approach on a RIL population (PIC-76, refer to Methods section for more details), derived from a cross between slow (PR9920–171) and fast-imbibing (TARS-HT1) genotypes, to identify the genomic regions associated with seed imbibition. Slow and fast imbibing pools, each containing 87 and 90 individuals, were sequenced to the depths of 116.7X and 110.7X, respectively. In total 332,689 SNPs were detected. SNP variation was not equally distributed among chromosomes (Table 1, Figure S3).

**Table 1:** Sequencing information of two pools used for Bulked Segregant Analysis

Chromosome	Size (bp)	Slow pool		Fast pool		Total marker ^b^
		Depth (X)	Coverage ^a^	Depth (X)	Coverage	
Pv01	51,432,818	111.9	96.9	105.8	97.0	6648
Pv02	49,674,440	143.2	97.4	129.2	97.6	25,592
Pv03	53,437,368	121.8	95.5	113.7	95.5	10,996
Pv04	48,050,588	105.0	89.7	100.7	89.7	1925
Pv05	40,922,943	99.0	93.9	97.4	93.9	34,441
Pv06	31,236,464	98.5	96.5	96.1	96.5	1783
Pv07	40,041,030	100.7	96.4	96.8	96.5	27,466
Pv08	63,043,462	92.2	89.4	91.1	89.6	13,755
Pv09	38,253,068	164.5	97.1	144.3	97.1	3104
Pv10	44,306,500	127.7	94.9	120.8	95.0	1478
Pv11	53,585,558	102.3	91.5	96.1	85.6	205,501
Genome	537,226,098	116.7	93.5	110.7	92.9	332,689

^a^ Sequencing coverage for regions that have between 40 and 500 reads

^b^ Total number of markers used for Bulked Segregant Analysis after filtering

**Fig. 4:**
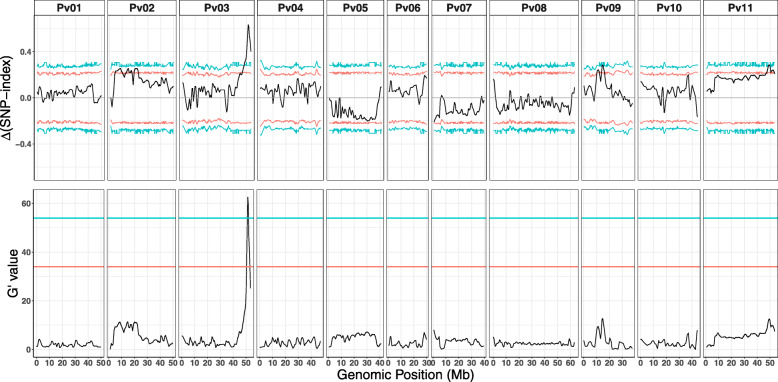
Genome-wide bulked segregant analysis detected a single major QTL at the end of Pv03. Two analytical methods were used for this analysis: the top figure represents the result of the ΔSNP-index and the bottom represents the G′ value. Red and blue lines indicate the 0.95 and 0.99 confidence intervals, respectively in the ΔSNP-index figure. In the G′ value figure, false discovery rates (FDR) of 0.05 and 0.01 are represented by red and blue threshold lines, respectively

Genome-wide BSA revealed a significant QTL in a 10 Mb interval between Pv03/43.1–53.4 Mb (FDR = 0.05), with the peak located at 51.73 Mb (Fig. 4). The peak of this QTL passed the 99% confidence interval threshold using the ΔSNP method and had a G-prime value of 65.9, indicating strong allele frequency differences between the two pools (Figure S3). Further analysis indicated that TARS-HT1 alleles within the major QTL on Pv03/43.1–53.4 Mbp were more frequent in the fast-imbibing pool and less frequent in slow-imbibing pool.

**Fig. 5:**
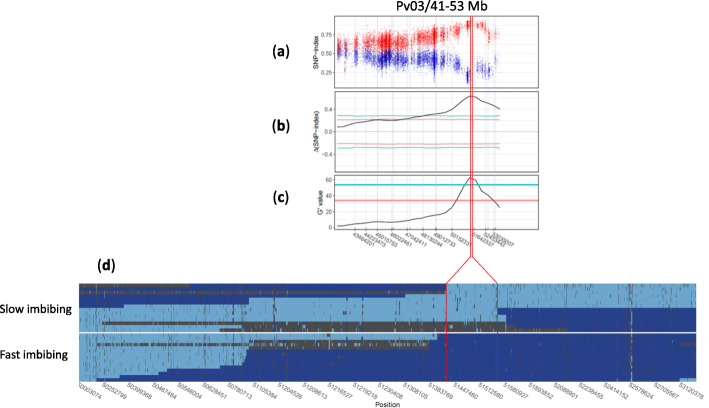
Fine mapping of the QTL at the end of Pv03. **a** The allele frequency difference between fast- and slow-imbibing pools at the QTL region. Each dot represents the TARS-HT1 allele frequency in the fast-imbibing (red) and slow-imbibing (blue) pools. **b**-**c** represent the detected QTL region using ΔSNP-index (**b**) and G′ (**c**) methods, respectively. **d** The haplotype map of 30 individuals that were whole-genome sequenced to narrow down the position of the QTL. The individuals were separated based on their water uptake phenotype. A 118 kbp region was detected (surrounded by red lines) that possessed PR9920–171 alleles (light blue) in slow imbibing genotypes. The same region had TARS-HT1 alleles (dark blue) for the fast imbibing genotypes. Three individuals were detected that were heterozygotes (dark gray) in the 118 kbp region and expressed the slow-imbibing phenotype. The red lines highlight the fine mapped region and its corresponding positions in the initial BSA

The fine-mapping of the QTL region was conducted on a subset of PIC-76 (derived from PR9920–171 × TARS-HT1, refer to Methods section for more details) containing 384 RILs. We, initially narrowed the QTL region down to a ~ 1.6 Mb interval, located between KASP-50.1 Mb and KASP-51.6 Mb (Figure S4). Then, we re-sequenced 30 individuals in which recombination events occurred between KASP-50.1 and KASP-51.6. This sequence data allowed us to screen the haplotype composition of the genomic region between 50 Mbp to the end of Pv03 (53.4 Mb). In total, 2460 SNPs were detected in this region. The whole genome sequencing of the fast- and slow-imbibing individuals revealed a ~ 118 Kb region between 51,426,054 and 51,544,057 (Fig. 5). Three individuals were heterozygous in this region and phenotypically grouped with slow-imbibing individuals. Within this ~ 118 Kb region, 11 genes were identified—including a tandem duplication of genes encoding a putative pectin acetylesterase 8 protein (Table 2).

**Table 2:** List of candidate genes within the QTL interval and their exonic polymorphisms between fast (TARS-HT1) and slow imbibing (PR9920–171) genotypes

Gene ID	Start	End	Arabidopsis homolog	Annotation	Abbreviation	Location	Indels ^a^	Point mutations ^b^
Phvul.003G277400	51,431,904	51,434,651	AT4G19440.1	Tetratricopeptide repeat -like	TPR-like	Chloroplast	–	1(T➔ M)
Phvul.003G277500	51,439,244	51,444,213	AT4G19420.1	Pectin acetylesterase 8	PAE-8-1	Extracellular region	–	–
Phvul.003G277600	51,454,061	51,458,474	AT4G19420.1	Pectin acetylesterase 8	PAE-8-2	Extracellular region	1(5 bp)	–
Phvul.003G277700	51,459,936	51,468,848	AT5G45290.1	RING/U-box superfamily		Nucleus	–	–
Phvul.003G277800	51,469,994	51,475,970	AT1G31650.1	RHO guanyl-nucleotide exchange factor 14		Mitochondrion	–	–
Phvul.003G277900	51,479,760	51,480,383	AT5G45320.1	Late embryogenesis abundant protein	LEA	Chloroplast	–	–
Phvul.003G278000	51,490,499	51,496,568	AT1G77360.1	*Arabidopsis* pentatricopeptide repeat 6	APPR-6	Mitochondrion	–	–
Phvul.003G278100	51,504,483	51,504,979	AT2G35612.1	Precursor of CEP4 – regulate nitrogen uptake		Extracellular region	–	–
Phvul.003G278200	51,514,163	51,522,692	AT4G19380.1	Long-chain fatty alcohol oxidase − 4	FAO-4	Chloroplast	–	4(G➔D, M➔L,I➔V, I➔F)
Phvul.003G278300	51,525,377	51,527,087	AT4G19370.1	MODIFYING WALL LIGNIN-2	MWL-2	Extracellular region	–	–
Phvul.003G278400	51,539,102	51,541,642	AT4G19230.1	Cytochrome P450–707 – A - polypeptide 1	CYP707A1	Chloroplast	–	1(P➔A)

^a^ Size of indels in bp is in parenthesis

^b^ The number of non-synonymous point mutations. The amino acid change is in parenthesis. There was no synonymous point mutation detected within these genes

### Coding sequence analysis of candidate genes

Seven variants were detected within exonic regions of four genes in the fine-mapped region. All of the point mutations within the exonic regions resulted in nonsynonymous substitutions. One nonsynonymous point mutation was detected in the only exon of *Phvul.003G277400*, which encodes for a Tetratricopeptide repeat. This point mutation results in an amino acid change (T➔M) at position 658 aa of the putative protein. We aligned the sequence of this protein with homologous ones and found that this change is located in a non-conserved region and thus, is unlikely to have profound functional effects (Figure S5).

Four non-synonymous point mutations were detected within exon 3 of *Phvul.003G278200*, which encodes a long-chain fatty alcohol oxidase − 4 (FAO-4, Figure S6). The aa change at position 275 (G➔D) and 500 (I➔F) were located within poorly conserved regions of this gene. The mutation at 443 aa (I➔V) is located within a conserved region. However, both PR9920–171 (I) and TARS-HT1 (V) alleles distributed equally among homologous genes, suggesting that both alleles are functional. We found that one aa change in FAO-4 at position 359 is at a more highly conserved position in the gene. In this position, TARS-HT1 coded for leucine (L), compared to methionine (M), which is the predominant aa at this position. However, both of these aa possess a hydrophobic side chain and it is unlikely that this substitution imposes a major modification in protein conformation.

Another non-synonymous point mutation (P➔A) was detected at the last exon (7th) of *Phvul.003G278400* at position 441 aa. This gene encodes a Cytochrome P450–707-A-polypeptide-1 (CYP707A1). We found that this mutation is located within a non-conserved region (Figure S7).

We identified a 5-bp insertion in the 6th exon of *Phvul.003G277600* in TARS-HT1, which is the second *pectin acetylesterase 8* (*PAE-8-2*). This insertion introduces a frameshift at 182 aa in the TARS-HT1 PAE-8 and results in introduction of an uncharacterized codon at 199 aa and potentially a truncated protein (Figure S8). Alignment of this gene with homologous genes in other species revealed that this frameshift mutation causes the loss of almost half of the protein including several conserved domains (Figure S8). We designed specific primers and amplified the indel site from cDNA template that confirms the expression of this region of the gene in both genotypes.

### Comparative expression analysis of candidate genes in the seed coat

**Fig. 6:**
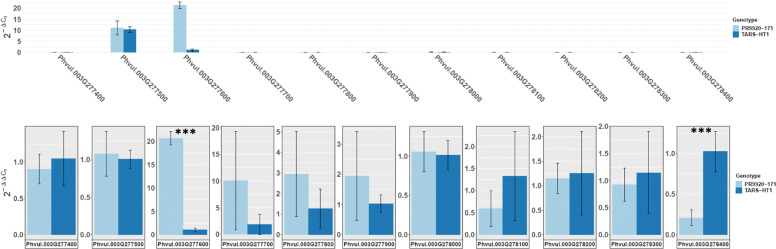
The expression pattern of eleven genes in the seed coat of PR9920–171 and TARS-HT1. The panel on the top shows the expression level of the 11 genes using 2^-ΔCt^. High expression was detected for *Phvul.003G277500* and *Phvul.003G277600*, which encode orthologs of for pectin acetylesterase 8. The bottom panel represents the 2^-ΔΔCt^ reflecting the relative expression fold change for each gene. Different relative expression was detected between PR9920–171 and TARS-HT1 for *Phvul.003G277600* and *Phvul.003G278400* (indicated by asterisks). Higher expression (~ 21 folds) of *Phvul.003G277600* was detected in the PR9920–171 seed coat relative to TARS-HT1. However, TARS-HT1 showed higher expression for *Phvul.003G278400* compared to PR9920–171. For both panels, dark and light blue represents TARS-HT1 and PR9920–171, respectively

To assess whether there is differential expression of candidate genes between PR9920–171 and TARS-HT1 seed coats, we performed RT-qPCR. This analysis revealed that *Phvul.003G277500* (in both genotypes) and *Phvul.003G277600* (in PR9920–171) are substantially expressed in the seed coat compared to other gene models (Fig. 6). Interestingly, both of these genes are homologous to the Arabidopsis gene *AT4G19420* that encodes pectin acetylesterase 8, which is expressed in extracellular regions. Although no expression differences were detected between genotypes for *Phvul.003G277500* (*PAE-8-1*), the expression level was about 21-fold lower in the TARS-HT1 seed coat for *Phvul.003G277600* (*PAE-8-2*). The 5-bp insertion in TARS-HT1 may have resulted in a post-transcriptional gene regulation that causes the lower stability of the PAE-8-2 transcript in TARS-HT1 seed coat.

### Differences in allele frequencies of PAE-8 between wild and domesticated beans

**Fig. 7:**
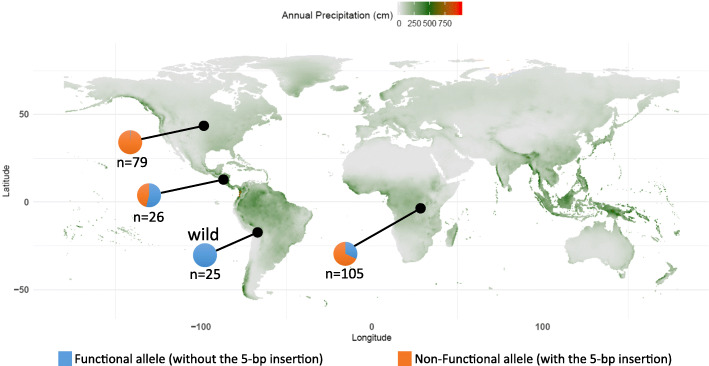
Allelic distribution of *Pectin acetylesterase 8–2* for the 5-bp indel. All of the wild Andean beans possessed the functional allele (without the 5-bp insertion). Among the domesticated beans, genotypes from North America were fixed for the non-functional allele (with the 5-bp insertion). Although beans originating from Middle America had almost equal portions of alleles, the non-functional allele was prevalent within bean populations originated from Africa. The historical (1970–2000) world annual precipitation data was acquired from WorldClim version 2 [41] and plotted by ggplot2 package [42] in R. The blue and orange in the pie charts indicate the proportions associated with the functional and non-functional alleles, respectively

We predicted that if a gene were involved in domestication, we would expect that allele frequencies of that gene would differ greatly between wild and domesticated bean populations. To test this prediction, we assessed the distribution of allele frequencies of the frameshift mutation allele within a subset of Andean Diversity Panel and in wild Andean beans (Fig. 7). This analysis revealed that all wild beans possessed the functional PAE-8-2 allele (PR9920–171 version, without the insertion). In contrast, the majority (77%) of domesticated genotypes have the non-functional allele with the 5-bp insertion (TARS-HT1 allele). This insertion was distributed differentially among geographical regions. The allele with 5-bp insertion was almost fixed (99%) in genotypes originating from North America. However, this allele was less frequent within genotypes collected from domesticated Andean beans in Africa (68%), and Middle America (46%).

## Discussion

In this study we found that water entry into the seed coat is initiated through lens structure on the seed. Genetic analysis revealed that a single major QTL at Pv03 underlies variation in seed coat impermeability. Based on fine-mapping, expression and haplotype analyses, we concluded that a 5-bp frameshift mutation in *pectin acetylesterase-8-2* is a candidate causal variant underlying this seed imbibition QTL. This frameshift mutation is absent within wild Andean beans, while being much more frequent within cultivated beans. Overall, these results suggest that human domestication of common bean contributed to strong selection for rapid seed water uptake that potentially caused a major shift in allele frequencies of the non-functional variant of a *pectin acetylesterase-8* ortholog.

### Importance of seed dormancy in beans

Seed dormancy in legumes, and in particular in common beans, is interwoven with their evolution and domestication [4]. Physical dormancy, in which seed coats are impermeable to water, is the most prevalent form of dormancy in legumes. In the current study, we showed that the absence of physical dormancy in a non-dormant genotype is associated with a rapid water uptake and respiration rate. If flooding (soaking) persists, the respiration rate decreases in non-dormant TARS-HT1 seeds compared to its earlier respirations, possibly due to earlier higher respiration and depletion of O_2_ in the environment. However, dormant PR9920–171 seeds maintain their respiration capacity for a longer period, which results in a higher germination (survival) rate when flooding ends. This indicates that physical dormancy can be beneficial in flooding stress tolerance. However, a low rate of seed imbibition is an undesirable trait in crop production as it decreases the synchronous germination and increases the cooking time [32]. As a consequence, the majority of domesticated beans in Andean Diversity Panel lack strong physical dormancy and therefore are susceptible to flooding at germination [35].

### Seed water uptake is initiated from the lens

Seed water uptake can occur through a number of different structures in common bean and the location of seed water uptake varies among genotypes [38]. In the genotypes evaluated here, we found that seed water uptake initiates in the lens. The lens structure is particularly important for imbibition in genotypes with the highest levels of physical dormancy. Further, micro-cracks in the lens groove are potentially the initial entry point for water into these seeds. Similarly, Hradilova et al. [5] reported more fissures in the lenses of non-dormant peas (*Pisum* spp.). Additional studies have found that the lens is the initial site of water entry in some Legume species [43–49]. However, seed water uptake mechanisms do vary among legume species [47].

### Seed dormancy is a part of domestication syndrome and is associated with a major QTL on Pv03

The domestication syndrome [50] is defined as a common set of phenotypic differences between cultivated crops and wild progenitors [3, 4]. In seed crops, loss of seed dormancy is often among the first traits selected during the first stage of domestication [3]. Several studies have shown that traits involved in the domestication syndrome are controlled by few loci of major effect [4, 19, 51, 52].

In common bean, several traits are involved in the domestication syndrome, including seed shattering, seed dormancy, determinacy, seed size, and photoperiod insensitivity [4, 53, 54]. Among these, seed dispersal (shattering) and dormancy are the most important traits distinguishing wild and domesticated beans [4]. Koinange [4] found that four unlinked loci controlled seed dormancy. The biggest effect (52% of the variance explained) QTL from their study was also located on Pv03. We blasted the QTL flanking markers from the Koinange study and were able to map it in an interval between 40.0 Mb and 53.2 Mb. In our study, a single QTL was detected between 51.4 and 51.5 Mb, and co-localized with the major QTL reported by Koinange et al. for seed dormancy. Identification of this major QTL controlling seed dormancy in two separate studies strongly suggests that seed water uptake is a key component of dormancy. Seed imbibition was also studied in two black bean populations and a major QTL on Pv07 was discovered [32, 55]. This is not unexpected because multiple lines of evidence have shown that overall domestication was associated with selection on different loci in the two gene pools [56], that flooding tolerance at germination stage is controlled by different loci in the domesticated gene pools of common beans [34, 35]. Other examples of distinct selection in the two gene pools was also observed for the determinacy *Fin* loci [57], and the master regulator of color expression gene *P* [58].

The major QTL detected in our study is located in the vicinity (~ 3.5 Mb) of a locus that controls pod shattering in common bean [54]. Genetic linkage between seed dormancy and pod shattering was also reported in lentils [19]. Co-localization of QTL controlling several domestication traits were reported in other non-legume crops, such as pearl millet [59] and rice [60]. Linkage between genes controlling important traits involved in domestication syndrome can be an important feature of domestication [3].

### A 5-bp insertion in pectin acetylestrase is likely the causative mutation for disruption of seed dormancy

The most functionally costly mutation in the coding region of candidate genes in the fine-mapped region of the seed water uptake QTL in our study was a 5-bp frameshift mutation in the *PAE-8-2* gene. Pectin plays a key role in cementing cells together [61]. Based on this hypothesis, pectin within the middle lamella are in a soluble form that can be depolymerized by high temperatures and/or water. When depolymerization occurs, cells can separate from each other. In insoluble forms, pectin is more resistant to water absorption that promotes middle lamellar stability and failure of cells to separate during water uptake. Kigel [62] suggested several mechanisms can contribute to pectin insolubility and the hard-to-cook phenomena in beans. Acetylation of pectin polymers has been shown to decrease the crosslinking of the pectates through calcium ions by steric hindrance consequently increases their solubility [63]. However, acetyl groups can be removed by *PAE* genes that contribute to cell wall hardiness [64]. Pectin deacetylation, by overexpression of *PAE*, reduces the digestibility of pectin polymers by pectinase [65]. Further, it has been shown that overexpression of mung bean (*Vigna radiate* L.) *PAE* in potato (*Solanum tuberosum* L.) results in potato tubers with stiffer cell walls [66]. The level of acetylation can also affect resistance to diseases [67] or gelling capacity of the pectin that is important in food processing [64].

**Fig. 8:**
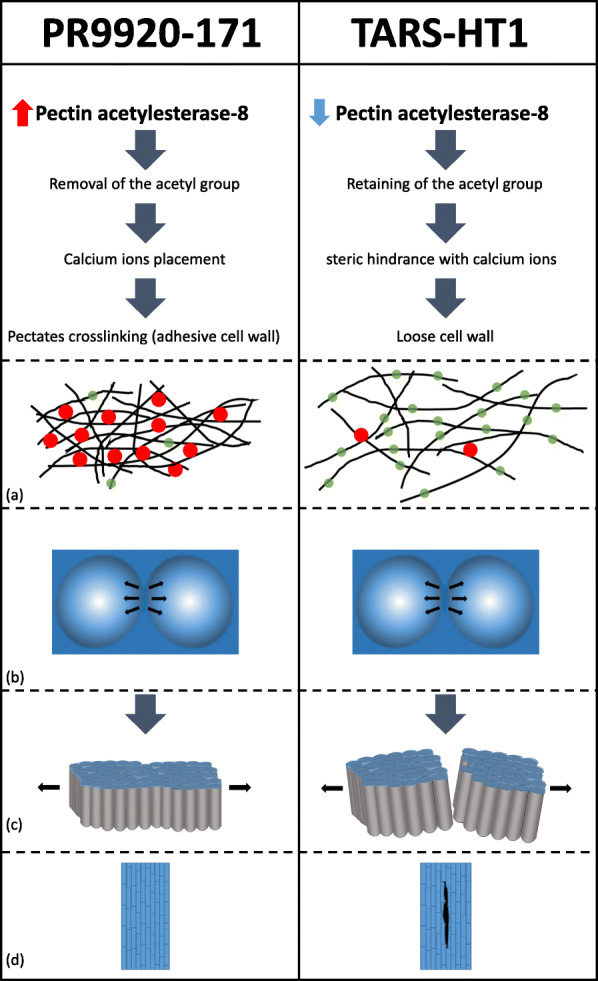
Hypothetical model of seed coat permeability in dormant (PR9920–171) and non-dormant (TARS-HT1) beans. Higher expression of pectin acetylesterase-8 in physically dormant seeds causes higher rate of acetyl group removal from the pectin polymers. This facilitates replacement of calcium ions and crosslinking of pectin polymers that convert pectin to an insoluble and strong form. Strong adhesion of pectin polymers resists separation of palisade cells against imposed tension of lens structure. However, in non-dormant seeds (TARS-HT1), a 5-bp insertion causes loss-of-function mutation in pectin acetylesterase-8-2. This loss-of-function result in remaining of acetyl groups that cause a steric hindrance with calcium ions. Lower calcium-mediated crosslinking among pectin polymers in this condition resulted in a looser cell wall that is more prone to the micro-fissures. Tension from swelling parts of lens causes the palisade cell in the lens groove to separate from each other and develop micro-cracks in loose non-dormant seed coat. **a** Schematic representation of pectin fibers within cell wall. Black solid lines represent pectin fibers. Green transparent circle represent acetyl groups and red circles represents calcium ions that facilitates crosslinking of pectin polymers. **b** Schematic representation of the lens on the seed coat. Black arrows indicate the possible tension imposed by the swelling structure of the lens on the lens groove cells. **c** Palisade cells located within the lens groove. Black lines indicate the possible tension imposed by the swelling structure of the lens on these cells that cause separation of cells in non-dormant seed coat. **d** The palisade cells separation in non-dormant seeds results in the appearance of micro-cracks within the lens groove

We hypothesize that higher expression of the *PAE-8* in PR9920–171 is associated with increased removal of acetyl groups in pectin, and consequently an increase in cell adhesion (Fig. 8). This hypothesis is consistent with our observations that TARS-HT1 has several micro-cracks within the lens groove, potentially due to non-functional PAE-8-2 and lower cell adhesion. This mechanism might be widespread within legumes, as it was observed that insoluble pectinaceous layers in palisade cells of wild *Pisum* spp. seeds were associated with water impermeability [23]. Further, it was proposed that pectin crosslinking properties are the major contributor of seed dormancy in wild *Pisum* spp. [5].

### Population genetic analyses suggest that PAE-8-2 was selected during domestication

The high allele frequency difference for *PAE-8-2* among wild and domesticated beans (Fig. 7) suggests that it was under strong selection by humans during domestication. The fact that we could not detect the non-functional allele of *PAE-8-2* within the wild beans screened in this study suggests that this allele arose from a new mutation during or post- domestication. While the non-functional allele of *PAE-8-2* was frequent in domesticated Andean beans, it was only near fixation for genotypes derived from Northern America. Andean beans from tropical regions (Africa and Middle America) retained the functional *PAE-8-2* allele at a moderate frequency. This observation suggests that there was a higher selection pressure for the non-functional allele in North America through modern breeding programs. It is plausible that faster imbibition and germination was positively selected in highly mechanized agricultural systems. Further, slow-imbibition may adversely affect bean quality. Therefore, alleles associated with insoluble pectin substances are predicted to be purged from germplasm by breeders.

The high level of seed dormancy in PR9920–171, which is a breeding line developed and tested in Puerto Rico [39] originated from IJR, which is a landrace grown in a tropical region of India. The functional PAE-8-2 allele in this landrace might contribute to higher environmental adaptability. It is likely that the functional allele of PAE-8-2 contributes to the insoluble pectin that can be beneficial in warm and humid conditions of tropical environments to prevent pre-harvest germination and/or promote seed survival during flooding. Physical dormancy may also be associated with higher quality of other legumes in the tropics, as increasing seed dormancy was suggested as a potential strategy to improve mung bean cultivars for cultivation in tropical environments [68].

## Conclusion

Physical seed dormancy is an important trait in domestication of many legume species, including common bean. This trait has been considered an undesirable factor in agricultural systems. However, this dogma has started to be questioned recently [9, 68]. The main contributor for this change in perspective is due to the unpredicted consequences of climate change. Recent shifts in precipitation patterns coincide with excess rain at planting, thus affecting germination or plant maturity, which results in reduced yield and seed quality. Consequently, the future trajectory of legume improvement should consider maintaining some level of physical dormancy, particularly in vulnerable flood-prone regions. To achieve this goal, more in-depth studies are necessary to uncover the genetic factors underlying this trait. In our current study, we found one major QTL associated with seed dormancy that is likely involved in bean domestication. This finding is consistent with what other research groups have proposed about domestication traits being controlled by few major genes. We also found that the likely causative mutation that facilitates bean domestication is a 5-bp insertion within *pectin acetylestrase-8* that may result in the loss of seed dormancy. Although our results provide strong evidences for the role of *pectin acetylestrase-8* in seed dormancy, further confirmations seems necessary by employing transgenic techniques.

## Methods

### Plant material

To identify the genetic basis of differential seed dormancy between PR9920–171 and TARS-HT1, we used a bi-parental population derived from PR9920–171 × TARS-HT1 (Figure S1). The segregating population and its parents were acquired from Dr. Timothy Porch and Dr. Phillip Miklas. The seed from F_1_ plants derived from the hybridization of PR9920–171 and TARS-HT1 were bulked and advanced in bulk each generation until the F_5_ generation as a part of the development of over 200 PIC (Phaseolus Improvement Cooperative; http://arsftfbean.uprm.edu/bean/?page_id=2) bulk breeding populations. This population has subsequently been named PIC-76. The bulking strategy that was used for development of PICs has the advantage of advancing more lines in each generation that can improve the level of captured diversity. However, it can skew the distribution of population due to effect of selection on seed number. In a bulked segregant analysis approach that was used herein (see below), such a skewed distribution does not affect the results since the tails of the population are being used for the analysis.

### Seed germination and imbibition assay

To establish the rate of seed germination and imbibition for the two genotypes, PR9920–171 and TARS-HT1, plants were grown in the greenhouse facility at Michigan State University from July to October 2019. The seeds were hand-harvested to prevent potential seed coat damage. Ten unscarified and scarified seeds from each genotype were pooled separately. Three sets of 10 seeds were evaluated in the germination and imbibition assay. To evaluate germination, seeds were placed between two wet cotton papers, germination percentages were evaluated every 8 hours started from 24 h after soaking. To evaluate the seed imbibition, dry seed weight was measured at the beginning of the study (time 0) and then beans were soaked. Water uptake rate was measured every hour for the first 10 hours, then every 8 h until 42 h. At each time point, water was drained, and excess water was removed by blotting with a cotton paper and then beans were weighted. The rate of water uptake for each time-point was calculated as

$$ Water\ uptake\ rate=\frac{seed\ weight\ after\ soaking- seed\ weight\ before\ soaking}{seed\ weight\ before\ soaking} $$

To test whether cotyledons of PR9920–171 and TARS-HT1 differed in their water uptake rate, seed coats were carefully removed using a razor blade. The cotyledons of exposed seeds were subjected to the same imbibition assay as for the intact seeds. Three sets of 10 seeds per genotype were screened with this assay. The water uptake rate was measured every hour for 8 h.

### Evaluation of phenotype consistency across diverse field environments

To understand how different environmental conditions affect the level of seed imbibition, PR9920–171 and TARS-HT1 genotypes were grown in three distinct field conditions in 2019 (Table S1). From each plot, four samples, each containing 10 seeds with intact seed coats, were randomly selected. After 6 hours of soaking in water, relative imbibition was measured using the above formula. IJR was included in the field trial in MI to compare its imbibition with PR9920–171 and TARS-HT1.

### Effect of physical dormancy on seed respiration

The respiration rate of seeds with intact and scarified coats was measured using a LI-6800 portable gas exchange system (LI-COR Bio-sciences, Lincoln, NE) with an insect respiration chamber attachment. Prior to soaking, each replicate (five seeds per replicate) was weighed and then soaked in distilled water. For intact seeds, the seeds were imbibed for 6, 24, 48, 72, 96 and 192 h, while scarified seeds were imbibed for 2, 6, 24, 48, and 72 h. At each measurement time point, seeds were patted dry with a paper towel, weighed, and placed inside the insect respiration chamber. The respiration rate was measured with the following parameters: 1000 μmol s^− 1^ flow rate, 70% air relative humidity, 400 μmol CO_2_, and 25 °C air temperature. Three technical measurements were conducted at 30 s intervals and were averaged to count as one biological measurement. Immediately after the respiration measurements, the seeds were placed back in water to continue the treatment until measurement at the next time point.

### Tracking water uptake into the seed

To identify the initial location of water entry into the seed coat, we employed imaging using a CT-scan with iodine solution as a contrasting agent. To the best of our knowledge, this is the first time CT-scan imaging system was used for tracking the water inside the seed. Although destructive staining systems were developed previously, the CT-scan system provides an opportunity to non-destructively track the water inside the seed in real time. Seeds from both genotypes were soaked in a Lugol solution (1.007 g/mL, Sigma-Aldrich, St. Louis, MO). At 30 min intervals after soaking, seeds were drained and screened using a NSI X3000 industrial CT X-ray scanner. The CT scanner was equipped with a 225 kV microfocus tube, tungsten target, and 8″ × 10″ detector (Varex 2520DX) with 1536 × 1920 pixel resolution. Scans were obtained as a continuous scan, consisting of 720 projections and 3 frame averages per projection. The tube voltage was set at 75 kV with an electron flux of 100 μA. Optical filters were not used during imaging. The projections were combined into a 3D CT image using efX-CT software from NSI (Rogers, Minnesota). Final voxel resolution was 75.9 μm.

### Scanning electron microscopy (SEM) sample preparation and images analysis

To detect any potential micro-structural differences between the lens (see results) of the two contrasting genotypes, we employed an SEM approach. Sample preparation and SEM were conducted at the Center of Advanced Microscopy at Michigan State University. Twenty seeds from each genotype (PR9920–171 and TARS-HT1) were mounted on the aluminum stubs using high vacuum carbon tabs (SPI supplies, West Chester, PA). The seeds were coated with osmium (~ 10 nm thickness) in an NEOC-AT osmium chemical vapor deposition coater (Meiwafosis Co. Osaka, Japan). The seeds were imaged in a JEOL 6610LV (tungsten hairpin emitter) scanning electron microscope (JEOL Ltd., Tokyo, Japan). ImageJ [69] was used to quantify the micro-crack area on the lens surface of seeds of both genotypes.

### Bulked segregant analysis (BSA) to understand the genetic architecture of seed imbibition

To identify the genomic locus (loci) associated with physical seed dormancy, we performed BSA on the PIC-76 RIL population. To ensure that seed coats of all experimental seeds were intact, we first examined the integrity of seed coat under a stereo microscope prior to the experiments. A total of 1000 seeds with intact seed coat were retained for imbibition experiments. Seeds were soaked in deionized water and kept in room temperature (22 °C). After 5 hours, 90 imbibing seeds from PIC-76 were planted in Suremix potting soil (SURE, Galesburg, MI, USA), while the rest of seeds remained under water. These 90 imbibed seeds were designated as the fast-imbibing pool. After 1 day, 87 seeds did not show any sign of imbibition. These seeds were considered as the slow-imbibing pool. We scarified all of the seeds from the slow-imbibing pool and planted them in the potting soil. After germination, leaf discs were collected from each individual of both fast and slow-imbibing beans and tissue from the disks was bulked together for each pool. The genomic DNA was extracted from bulked tissue from both pools and parental lines using a Qiagen DNeasy Plant Mini Kit (Hilden, Germany).

Library preparation and sequencing was conducted at Michigan State University Genomics Core (https://rtsf.natsci.msu.edu/genomics/). Briefly, libraries were prepared using Illumina TruSeq Nano DNA library preparation kit following manufacturer recommendations. Completed libraries were QC’d and quantified using a combination of Qubit dsDNA HS (Invitrogen INC. Carlsbad, CA) and Advanced Analytical Fragment Analyzer High Sensitivity DNA assays. The sequencing was performed using the Illumina HiSeq 4000 platform in a 2 × 150 bp paired-end format. The raw reads were trimmed using the Cutadapt (v1.14 [70];). First, 15 bp of the reads from the 5′ end were removed from paired reads and then the bases with quality of less than 20 were trimmed from both the 5′ and 3′ ends. FastQC (v0.11.3) was performed to confirm the quality of the reads and removal of the adaptors. Reads were aligned to the *P. vulgaris* reference genome (v 2.1, [56]) using the BWA-MEM algorithm [71]. BAM files were sorted using SAMtools [72]. To remove the duplicates, we performed the Picard MarkDuplicates function [73]. This function identifies PCR and optical duplicates. We followed the GATK4 protocol to call SNPs and measured the allele frequency within each pool. SNPs were called using the HaplotypeCaller [74]. Only SNPs with a total number of reads higher than 40 and lower than 500 were retained for the downstream analysis. To further remove the unreliable variants, only SNPs that both alleles were detected in either parents (PR9920–171 and TARS-HT1) were retained in the analysis. Position and allele frequency of each SNP in each pool was retrieved using VariantsToTable in the GATK package. The table containing allele frequency data was imported into the R environment [75] and downstream analysis was performed using QTLseqr package [76]. QTL were identified with both the G prime and QTLseq methods. For both functions, the window size was set to 1 Mbp. The QTL intervals were defined by the first and last markers that passed the FDR threshold of 0.05.

### Fine mapping the seed imbibition QTL

To fine-map the seed dormancy QTL identified by the bulked segregant analysis, we saturated the genomic region surrounding the QTL. Initially, two Insertion/Deletion (InDel) markers and two KASP (Kompetitive allele specific PCR) markers were designed on the boundaries of the QTL on Pv03 (Table S2). In addition, another KASP marker was designed at 51.6 Mbp (the QTL peak) to detect possible double recombinant genotypes. A total of 384 individuals of PIC-76 were screened for these three markers to identify individuals with recombination in this region. Eighty-four recombinant individuals were identified and their seeds were sown in one-gallon pots. An additional 13 KASP markers were developed across the QTL to saturate the region (Table S2). At maturity, seeds were hand-harvested from 84 recombinant individuals. Six seeds from each plant were soaked in Lugol solution (Sigma-Aldrich, St. Louis, MO) for 4 hours at 22 C and visualized in the X-ray CT scanner (see above). To narrow-down the location of the gene associated with the seed imbibition we used QTL-Cartographer [77]. To further narrow-down the position of the QTL, 30 recombinant plants were re-sequenced (10X) to identify the exact position of recombination breakpoints. Sample libraries were prepared using the AgSeq pipeline that was developed at Texas A&M AgriLife Genomics and Bioinformatics Service. Libraries were then sequenced using the Illumina NovaSeq at Texas A&M AgrLife Research. Sequence cluster identification, quality pre-filtering, base calling and uncertainty assessment were done in real time using Illumina’s NCS 1.0.2 and RFV 1.0.2 software with default parameter settings.

### Haplotype survey within Andean cultivars and wild beans

Fine-mapping and detailed sequence analysis resulted in the identification of a likely candidate gene controlling variation in seed imbibition (see results). To test the hypothesis that this gene was involved in the process of domestication, we compared the haplotype frequencies between wild and domesticated Andean beans. Domesticated Andean beans were acquired from Dry Bean Breeding and Genetics Program at Michigan State University. Wild Andean beans were acquired from USDA-ARS Germplasm Resources Information Network (GRIN). We first surveyed 210 genotypes (Table S3) belonging to the Andean Diversity Panel [78], which were genotyped for a 5-bp indel that was detected in *PAE-8-2* (see below). These genotypes were collected from three distinct geographical regions; Africa (*n* = 105), North America (*n* = 79), and Middle America (Caribbean, Central America and Colombia, *n* = 26). In addition, we genotyped the same variant in 25 wild beans collected from the Andes region of South America (Table S4).

### Expression comparison of candidate genes between contrasting genotypes

To compare the expression profile of the genes within the fine-mapped region, we performed an expression analysis. Plants of both PR9920–171 and TARS-HT1 were grown at 25/20 °C (day and night temperatures, respectively) in a growth chamber (Big foot, Biochambers, Winnipeg, MB, Canada). At the flowering stage, the date of anthesis was recorded for each flower, separately. Seeds were collected at 22 days after pollination (early pod filling stage). Seed coats were separated from other seed parts and flash frozen using liquid nitrogen. The seed coat tissues of five seeds were pooled together for each biological replication. Four biological replicates were used in this experiment. Total RNA was extracted using the Spectrum Plant Total RNA Kit (Sigma-Aldrich, St. Louis, MO) following the manufacturer protocol. To remove any trace of genomic DNA, on-column DNase digestion was performed. The quantity and quality of extracted RNA was checked using a Qubit fluorometer (Invitrogen INC. Carlsbad, CA) and 1% Agarose gel, respectively. Reverse transcriptase and qPCR reactions were performed using Go-Taq 2-Step RT-qPCR System (Promega, Madison, WI) following the manufacturer’s protocol. For each biological replication, −RT reactions were included as negative controls to check for presence of any genomic DNA. Synthesized cDNA was diluted in 1:20 ratio. Eleven primer sets (Table S2) were designed for each of the candidate genes within the QTL interval. The primers were designed from conserved exonic sequences between both genotypes. Actin-11 (*Phvul.008G011000*) was used as the reference gene for qPCR expression analysis. Actin-11 was reported to have consistent expression under both biotic and abiotic stresses in common bean [79]. qPCR was performed using a Bio-Rad CFX384 Real-Time System (Hercules, CA). Four biological and two technical replicates were evaluated for each genotype. We used 2^−*ΔΔCt*^ method to identify the differentially expressed genes between the two genotypes.

## Supplementary Information

**Additional file 1: Figure S1.** Pedigree of the PR9920-171 and TARS-HT1. **Figure S2.** The effect of lens blockage on seed imbibition. **Figure S3.** The allele frequency of SNP markers within fast and slow imbibing pools. **Figure S4.** The saturation of QTL region with 18 KASP and Indel markers **Figure S5.** The conservation of the non-synonymous point mutation in the *Phvul.003G277400* coding region among homologous genes in other species. **Figure S6.** The conservation of the non-synonymous point mutations in the *Phvul.003G278200* coding region among homologous genes in other species. **Figure S7.** The non-synonymous point mutation in the *Phvul.003G278400* coding region. **Figure S8.** A 5-bp insertion in 6th exon of *Phvul.003G277600* causes a frameshift in the aa sequence of TARS-HT1.

**Additional file 2: Table S1.** Field sites information

**Additional file 3: Table S2** List of primers used in this study

**Additional file 4: Table S3.** List of domesticated Andean genotypes used in this study

**Additional file 5: Table S4.** List of wild Andean beans used in this study

## Data Availability

The sequence datasets generated during the current study are available in the NCBI Sequence Read Archive (SRA) repository, under BioProject ID: PRJNA666967.

## Abbreviations

aa: Amino Acid
ABA: Abscisic Acid
BSA: Bulked segregant analysis
CT-scan: Computerized Tomography Scan
GA: Gibberellins
IJR: Indeterminate Jamaica Red
Mbp: Mega base pairs
PAE-8-2: Pectin acetylesterase 8
SEM: Scanning electron microscopy

## References

[1] Diamond J (2002). Evolution, consequences and future of plant and animal domestication. Nature.

[2] Purugganan MD (2019). Evolutionary insights into the nature of plant domestication. Curr Biol.

[3] Meyer RS, Purugganan MD (2013). Evolution of crop species: genetics of domestication and diversification. Nat Rev Genet.

[4] Koinange EMK, Singh SP, Gepts P (1996). Genetic control of the domestication syndrome in common bean. Crop Sci.

[5] Hradilová I, Trněný O, Válková M, Cechová M, Janská A, Prokešová L (2017). A combined comparative transcriptomic, metabolomic, and anatomical analyses of two key domestication traits: pod dehiscence and seed dormancy in pea (*Pisum* sp.). Front Plant Sci.

[6] Rau D, Murgia ML, Rodriguez M, Bitocchi E, Bellucci E, Fois D (2019). Genomic dissection of pod shattering in common bean: mutations at non-orthologous loci at the basis of convergent phenotypic evolution under domestication of leguminous species. Plant J.

[7] Simpson KJ, Wade RN, Rees M, Osborne CP, Hartley SE (2017). Still armed after domestication? Impacts of domestication and agronomic selection on silicon defences in cereals. Funct Ecol.

[8] Moreira X, Abdala-Roberts L, Gols R, Francisco M (2018). Plant domestication decreases both constitutive and induced chemical defences by direct selection against defensive traits. Sci Rep.

[9] Smýkal P, Vernoud V, Blair MW, Soukup A, Thompson RD (2014). The role of the testa during development and in establishment of dormancy of the legume seed. Front Plant Sci.

[10] Finch-Savage WE, Footitt S (2017). Seed dormancy cycling and the regulation of dormancy mechanisms to time germination in variable field environments. J Exp Bot.

[11] Penfield S (2017). Seed dormancy and germination. Curr Biol.

[12] Ladizinsky G (1987). Pulse domestication before cultivation. Econ Bot.

[13] Abbo S, Zezak I, Schwartz E, Lev-Yadun S, Gopher A (2008). Experimental harvesting of wild peas in Israel: implications for the origins of near east farming. J Archaeol Sci.

[14] Gubler F, Millar AA, Jacobsen JV (2005). Dormancy release, ABA and pre-harvest sprouting. Curr Opin Plant Biol.

[15] Ahmad S, Khulbe RK, Roy D (2014). Evaluation of mungbean (*Vigna radiata*) germplasm for pre-harvest sprouting tolerance. Legum Res.

[16] Finch-Savage WE, Leubner-Metzger G (2006). Seed dormancy and the control of germination. New Phytol.

[17] Martin AC (1946). The comparative internal morphology of seeds. Am Midl Nat.

[18] Forbes I, Wells HD (1968). Hard and soft seededness in blue lupine, *Lupinus angustifolius* L.: inheritance and phenotype classification. Crop Sci.

[19] Ladizinsky G (1985). The genetics of hard seed coat in the genus *Lens*. Euphytica.

[20] Isemura T, Kaga A, Tomooka N, Shimizu T, Vaughan DA (2010). The genetics of domestication of rice bean, *Vigna umbellata*. Ann Bot.

[21] Isemura T, Kaga A, Tabata S, Somta P, Srinives P, Shimizu T (2012). Construction of a genetic linkage map and genetic analysis of domestication related traits in mungbean (*Vigna radiata*). PLoS One.

[22] Kongjaimun A, Kaga A, Tomooka N, Somta P, Vaughan DA, Srinives P (2012). The genetics of domestication of yardlong bean, *Vigna unguiculata* (L.) Walp. Ssp. unguiculata cv.-gr. Sesquipedalis. Ann Bot.

[23] Werker E, Marbach I, Mayer AM (1979). Relation between the anatomy of the testa, water permeability and the presence of phenolics in the genus *Pisum*. Ann Bot.

[24] Kannenberg LW, Allard RW (1964). An association between pigment and lignin formation in the seed coat of the lima bean. Crop Sci.

[25] Shao S, Meyer CJ, Ma F, Peterson CA, Bernards MA (2007). The outermost cuticle of soybean seeds: chemical composition and function during imbibition. J Exp Bot.

[26] Bradford K, Nonogaki H (2007). Seed development, dormancy and germination.

[27] Esau K (1965). Plant Anatomy.

[28] Ma F, Cholewa E, Mohamed T, Peterson CA, Gijzen M (2004). Cracks in the palisade cuticle of soybean seed coats correlate with their permeability to water. Ann Bot.

[29] Bitocchi E, Nanni L, Bellucci E, Rossi M, Giardini A, Zeuli PS (2012). Mesoamerican origin of the common bean (*Phaseolus vulgaris* L.) is revealed by sequence data. Proc Natl Acad Sci U S A.

[30] Gepts P, Osborn TC, Rashka K, Bliss FA (1986). Phaseolin-protein variability in wild forms and landraces of the common bean (*Phaseolus vulgaris*): evidence for multiple centers of domestication. Econ Bot.

[31] Emiliano T, Andrea B, Martina L, Alice I, Stefania V, Laura N, et al. Ancient genomes reveal early Andean farmers selected common beans while preserving diversity. bioRxiv. 2019:791806.

[32] Cichy KA, Fernandez A, Kilian A, Kelly JD, Galeano CH, Shaw S (2014). QTL analysis of canning quality and color retention in black beans (*Phaseolus vulgaris* L.). Mol Breed.

[33] Hosfield GL (1991). Genetic control of production and food quality factors in dry bean. Food Technol.

[34] Soltani A, MafiMoghaddam S, Walter K, Restrepo-Montoya D, Mamidi S, Schroder S (2017). Genetic architecture of flooding tolerance in dry bean middle-american diversity panel. Front Plant Sci.

[35] Soltani A, MafiMoghaddam S, Oladzad-Abbasabadi A, Walter K, Kearns PJ, Vasquez-Guzman J (2018). Genetic analysis of flooding tolerance in an andean diversity panel of dry bean (*Phaseolus vulgaris* L.). Front Plant Sci.

[36] Koornneef M, Bentsink L, Hilhorst H (2002). Seed dormancy and germination. Curr Opin Plant Biol.

[37] Stanley DW (1992). Hard beans— a problem for growers, processors, and consumers. Am Soc Hortic Sci.

[38] Agbo G, Hosfield G, Uebersax M, Klomparens K (1987). Seed microstructure and its relationship to water uptake in isogenic lines and a cultivar of dry beans (*Phaseolus vulgaris* L.). Food Struct.

[39] Román-Avilés B, Beaver JS (2003). Inheritance of heat tolerance in common bean of Andean origin. J Agric Univ Puerto Rico.

[40] Porch TG, Smith JR, Beaver JS, Griffiths PD, Canaday CH (2010). TARS-HT1 and TARS-HT2 heat-tolerant dry bean germplasm. HortScience.

[41] Fick S, Hijmans R (2017). WorldClim 2: new 1-km spatial resolution climate surfaces for global land areas. Int J Climatol.

[42] Wickham H (2016). ggplot2: elegant graphics for data analysis.

[43] Baskin JM, Baskin CC (2000). Evolutionary considerations of claims for physical dormancy-break by microbial action and abrasion by soil particles. Seed Sci Res.

[44] Baskin CC (2003). Breaking physical dormancy in seeds - focusing on the lens. New Phytol.

[45] Karaki T, Watanabe Y, Kondo T, Koike T (2012). Strophiole of seeds of the black locust acts as a water gap. Plant Species Biol.

[46] Hu XW, Wang YR, Wu YP, Baskin CC (2009). Role of the lens in controlling water uptake in seeds of two Fabaceae (*Papilionoideae*) species treated with sulphuric acid and hot water. Seed Sci Res.

[47] Morrison D, McClay K, Porter C, Rish S (1998). The role of the lens in controlling heat-induced breakdown of testa-imposed dormancy in native Australian legumes. Ann Bot.

[48] Jaganathan GK, Li J, Biddick M, Han K, Song D, Yang Y (2019). Mechanisms underpinning the onset of seed coat impermeability and dormancy-break in *Astragalus adsurgens*. Sci Rep.

[49] Yousif MAI, Wang YR, Dali C (2020). Seed dormancy overcoming and seed coat structure change in *Leucaena leucocephala* and *Acacia nilotica*. Forest Sci Technol.

[50] Hammer K (1984). The domestication syndrome (in German.). Die Kult.

[51] Fatokun CA, Menancio-Hautea DI, Danesh D, Young ND (1992). Evidence for orthologous seed weight genes in cowpea and mung bean based on RFLP mapping. Genetics.

[52] Doebley J, Stec A (1993). Inheritance of the morphological differences between maize and teosinte: comparison of results for two F2 populations. Genetics.

[53] Vittori V, Bitocchi E, Rodriguez M, Alseekh S, Bellucci E, Nanni L, et al. Pod indehiscence in common bean is associated to the fine regulation of PvMYB26 and a non-functional abscission layer. bioRxiv. 2020.

[54] Parker TA, Palkovic A, Jernstedt J, Gepts P (2020). Pod indehiscence is a domestication and aridity resilience trait in common bean. New Phytol.

[55] Sandhu KS, You FM, Conner RL, Balasubramanian PM, Hou A (2018). Genetic analysis and QTL mapping of the seed hardness trait in a black common bean (*Phaseolus vulgaris*) recombinant inbred line (RIL) population. Mol Breed.

[56] Schmutz J, McClean PE, Mamidi S, Wu GA, Cannon SB, Grimwood J (2014). A reference genome for common bean and genome-wide analysis of dual domestications. Nat Genet.

[57] Repinski SL, Gepts P (2012). The common bean growth habit gene PvTFL1y is a functional homolog of Arabidopsis TFL1. Theor Appl Genet.

[58] McClean PE, Bett K, Stonehouse R, Lee R, Pflieger S, MafiMoghaddam S (2018). White seed color in common bean (*Phaseolus vulgaris*) results from convergent evolution in the P (pigment) gene. New Phytol.

[59] Poncet V, Martel E, Allouis S, Devos K, Lamy F, Sarr A (2002). Comparative analysis of QTLs affecting domestication traits between two domesticated × wild pearl millet (*Pennisetum glaucum* L., Poaceae) crosses. Theor Appl Genet.

[60] Cai H, Morishima H (2002). QTL clusters reflect character associations in wild and cultivated rice. Theor Appl Genet.

[61] Galiotou-Panayotou M, Kyriakidis NB, Margaris I (2008). Phytase–phytate–pectin hypothesis and quality of legumes cooked in calcium solutions. J Sci Food Agric.

[62] Kigel J (1999). Culinary and nutritional quality of *Phaseolus vulgaris* seeds as affected by environmental factors. BASE.

[63] Ralet M-C, Crépeau M-J, Buchholt H-C, Thibault J-F (2003). Polyelectrolyte behaviour and calcium binding properties of sugar beet pectins differing in their degrees of methylation and acetylation. Biochem Eng J.

[64] de Souza A, Hull PA, Gille S, Pauly M (2014). Identification and functional characterization of the distinct plant pectin esterases PAE8 and PAE9 and their deletion mutants. Planta.

[65] Gou J-Y, Miller LM, Hou G, Yu X-H, Chen X-Y, Liu C-J (2012). Acetylesterase-mediated deacetylation of pectin impairs cell elongation, pollen germination, and plant reproduction. Plant Cell.

[66] Orfila C, Degan FD, Jørgensen B, Scheller HV, Ray PM, Ulvskov P (2012). Expression of mung bean pectin acetyl esterase in potato tubers: effect on acetylation of cell wall polymers and tuber mechanical properties. Planta.

[67] Pogorelko G, Lionetti V, Fursova O, Sundaram RM, Qi M, Whitham SA (2013). Arabidopsis and *Brachypodium distachyon* transgenic plants expressing *Aspergillus nidulans* acetylesterases have decreased degree ofpolysaccharide acetylation and increased resistance to pathogens. Plant Physiol.

[68] Humphry ME, Lambrides CJ, Chapman SC, Aitken EAB, Imrie BC, Lawn RJ (2005). Relationships between hard-seededness and seed weight in mungbean (*Vigna radiata*) assessed by QTL analysis. Plant Breed.

[69] Schneider CA, Rasband WS, Eliceiri KW (2012). NIH image to ImageJ: 25 years of image analysis. Nat Methods.

[70] Martin M (2011). Cutadapt removes adapter sequences from high-throughput sequencing reads. EMBnet J.

[71] Li H, Durbin R (2009). Fast and accurate short read alignment with burrows-wheeler transform. Bioinformatics.

[72] Li H, Handsaker B, Wysoker A, Fennell T, Ruan J, Homer N (2009). The sequence alignment/map format and SAMtools. Bioinformatics.

[73] Broad Institute (2019). Picard toolkit.

[74] Poplin R, Ruano-Rubio V, DePristo MA, Fennell TJ, Carneiro MO, Van der Auwera GA, et al. Scaling accurate genetic variant discovery to tens of thousands of samples. bioRxiv. 2018:201178.

[75] R Development Core Team R (2011). R: a language and environment for statistical computing. R Foundation for Statistical Computing.

[76] Mansfeld BN, Grumet R (2018). QTLseqr: an R package for bulk segregant analysis with next-generation sequencing. Plant Genome.

[77] Wang S, Basten CJ, Zeng Z (2012). Windows QTL Cartographer 2.5.

[78] Cichy KA, Porch TG, Beaver JS, Cregan P, Fourie D, Glahn RP (2015). A *Phaseolus vulgaris* diversity panel for andean bean improvement. Crop Sci.

[79] Borges A, Tsai SM, Caldas DGG (2012). Validation of reference genes for RT-qPCR normalization in common bean during biotic and abiotic stresses. Plant Cell Rep.

