# Supplementary material for: The genetics and physiology of seed dormancy, a crucial trait in common bean domestication
Source: BMC Plant Biol. 2021 Jan 22;21:58. doi: 10.1186/s12870-021-02837-6 (PMC7821524; doi:10.1186/s12870-021-02837-6)
Supplement: Supplementary file 1 — Additional file 1: Figure S1. Pedigree of the PR9920-171 and TARS-HT1. Figure S2. The effect of lens blockage on seed imbibition. Figure S3. The allele frequency of SNP markers within fast and slow imbibing pools. Figure S4. The saturation of QTL region with 18 KASP and Indel markers Figure S5. The conservation of the non-synonymous point mutation in the Phvul.003G277400 coding region among homologous genes in other species. Figure S6. The conservation of the non-synonymous point mutations in the Phvul.003G278200 coding region among homologous genes in other species. Figure S7. The non-synonymous point mutation in the Phvul.003G278400 coding region. Figure S8. A 5-bp insertion in 6th exon of Phvul.003G277600 causes a frameshift in the aa sequence of TARS-HT1. [file 12870_2021_2837_MOESM1_ESM.docx]

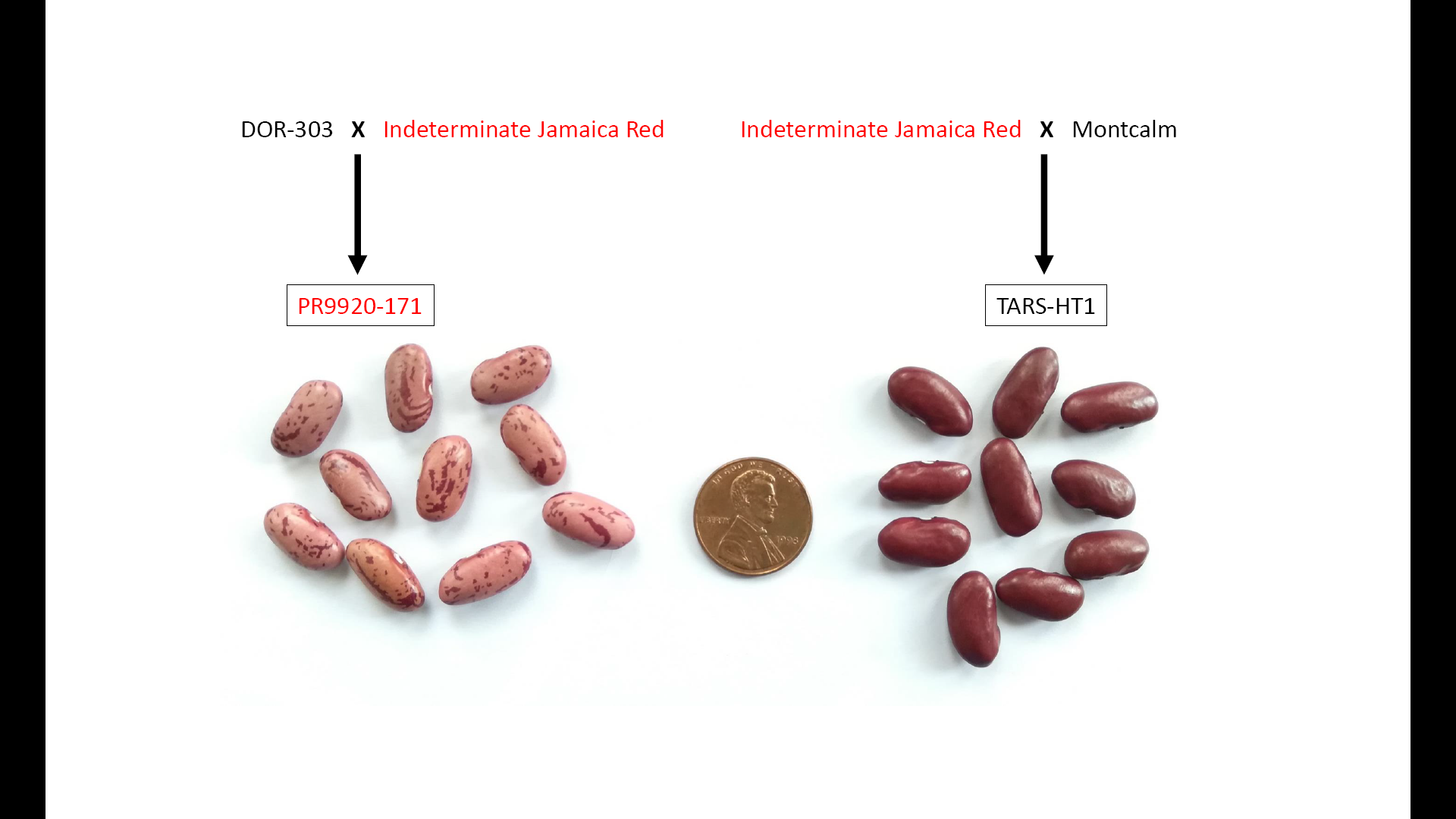


Figure S1. Pedigree of the PR9920-171 and TARS-HT1 that were used in this study. Both genotypes shared Indeterminate Jamaica Red (IJR) as a parent. IJR and PR9920-171 are both slow-imbibing beans. Genotypes with the slow-imbibition phenotype were highlighted by red.


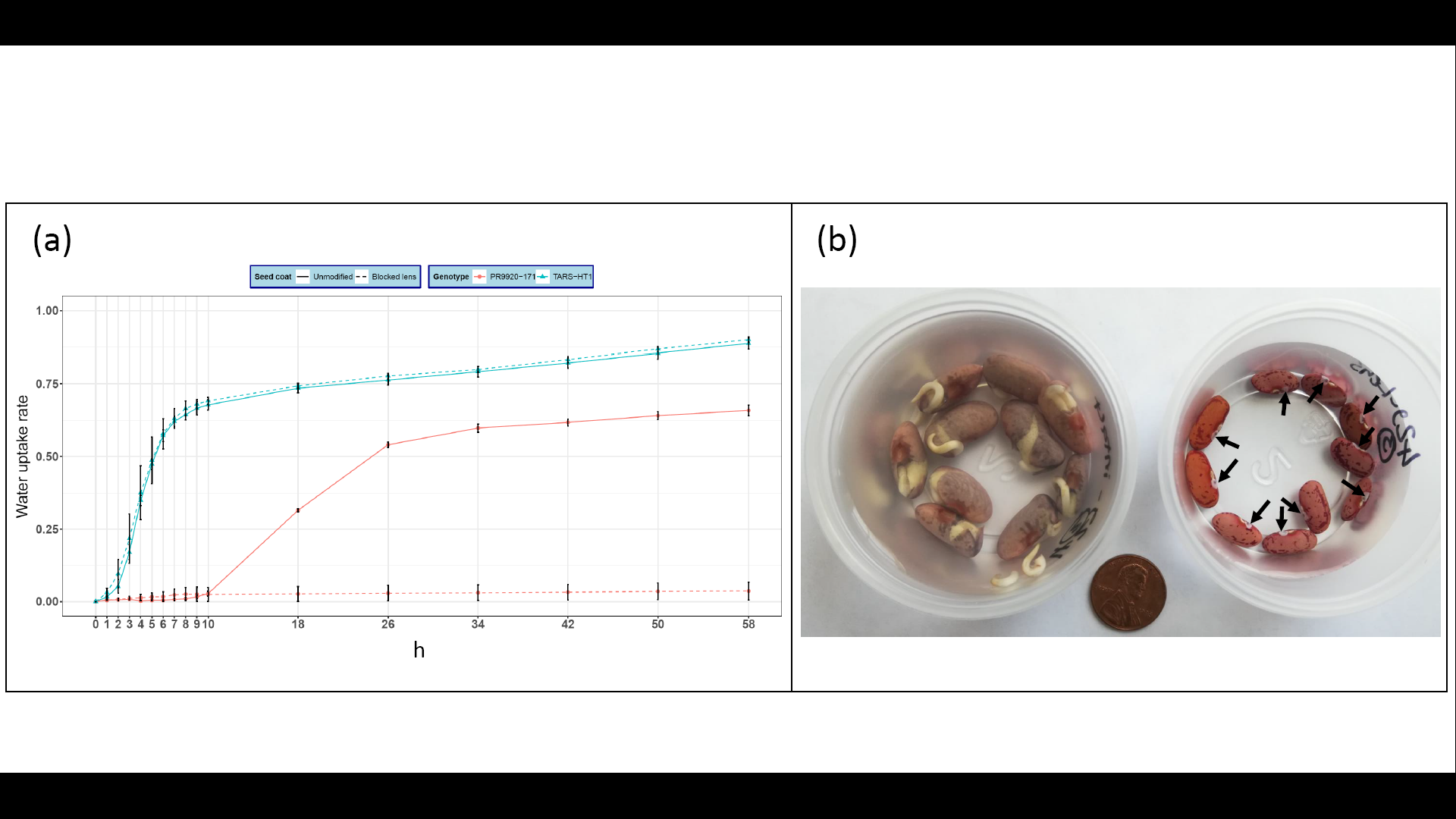


Figure S2. The effect of lens blockage on seed imbibition. a) The TARS-HT1 seeds (blue), with blocked lenses, followed a similar imbibition pattern as the un-modified (natural) seed. In contrast, blocking the PR9920-171 (red) lenses completely prevented imbibition. This indicates that the lens is a crucial structure for water imbibition for PR9920-171. b) Comparison of PR9920-171 seeds with an unmodified seed coat (left) and PR9920-171 seeds with blocked lenses (right) after one week of soaking. Blocking the lens with nail polish completely prevented seed imbibition. Arrows indicate the blocked lens with nail polish.


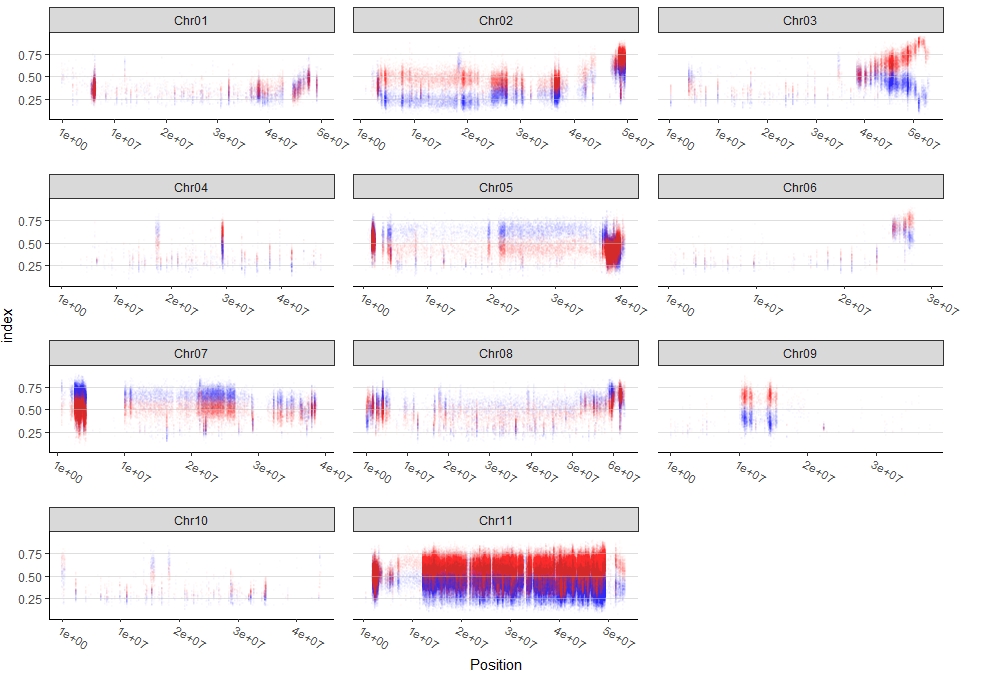


Figure S3. The allele frequency of SNP markers within fast and slow imbibing pools derived from a segregating population (PR9920-171 × TARS-HT1). Dots represents the TARS-HT1 allele frequency in fast- (red) and slow- (blue) imbibing pools.


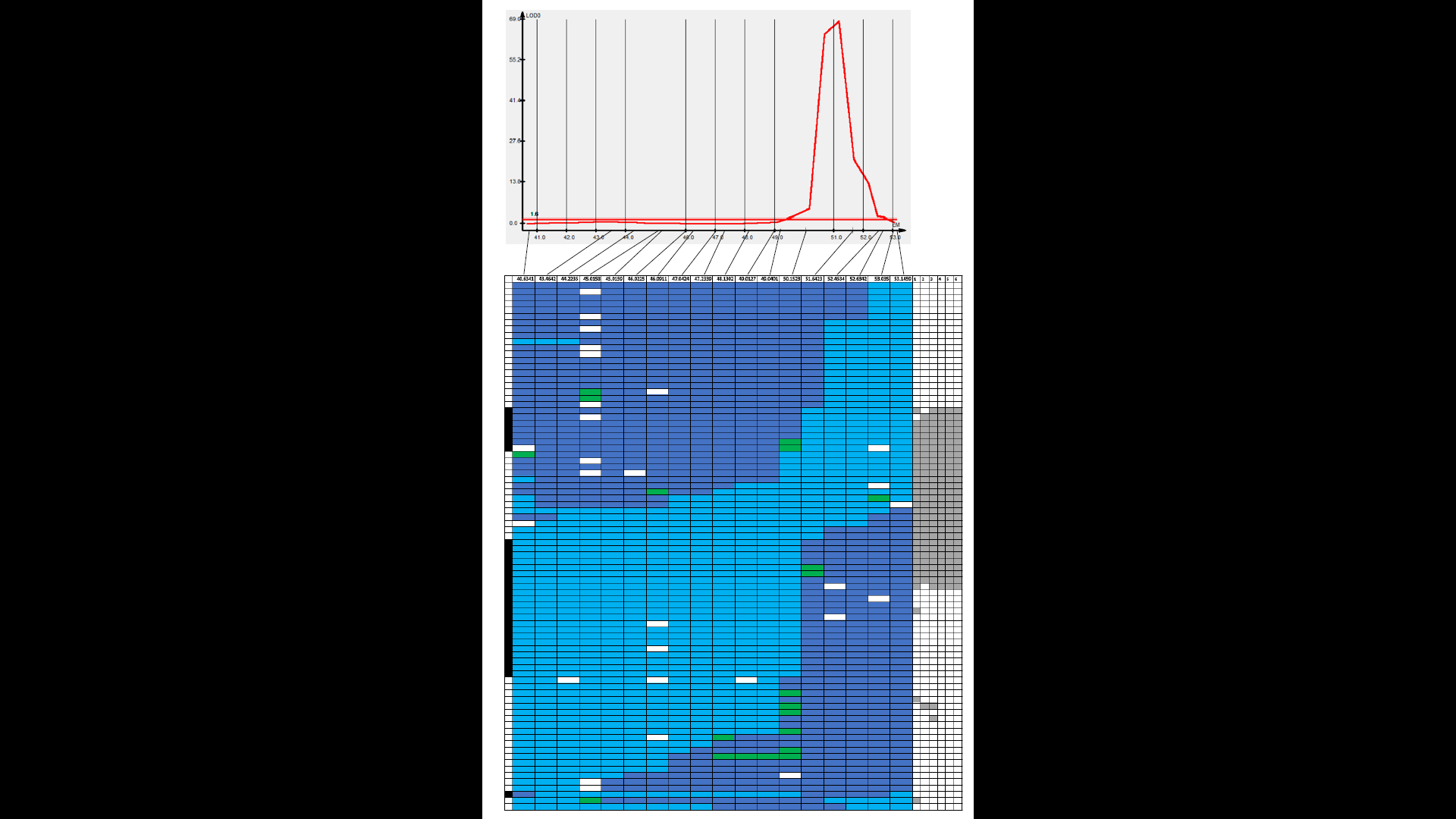


Figure S4. The saturation of QTL region with 18 KASP and Indel markers. The result of QTL-cartographer on the top revealed a significant peak (LOD = ~69) between two markers at positions 50.1 Mb and 51.6 Mb. The figure on the bottom represents the haplotype map of 84 individuals (rows) that showed evidence of recombination within the initial QTL. Dark and light blue represents allele originated from TARS-HT1 or PR9920-171, respectively. Heterozygotes and un-characterized positions were indicated by green and white cells, respectively. The black cells in the first column highlight the 30 individuals that showed evidence of recombination between markers at 50.1 and 51.6 Mb. These individual’s whole genomes were sequenced to identify the exact position of recombination (refer to figure 5). The last six rows indicates the imbibition status of the corresponding individuals seeds, as captured by CT-scan imaging. Grey cells indicate no water uptake.


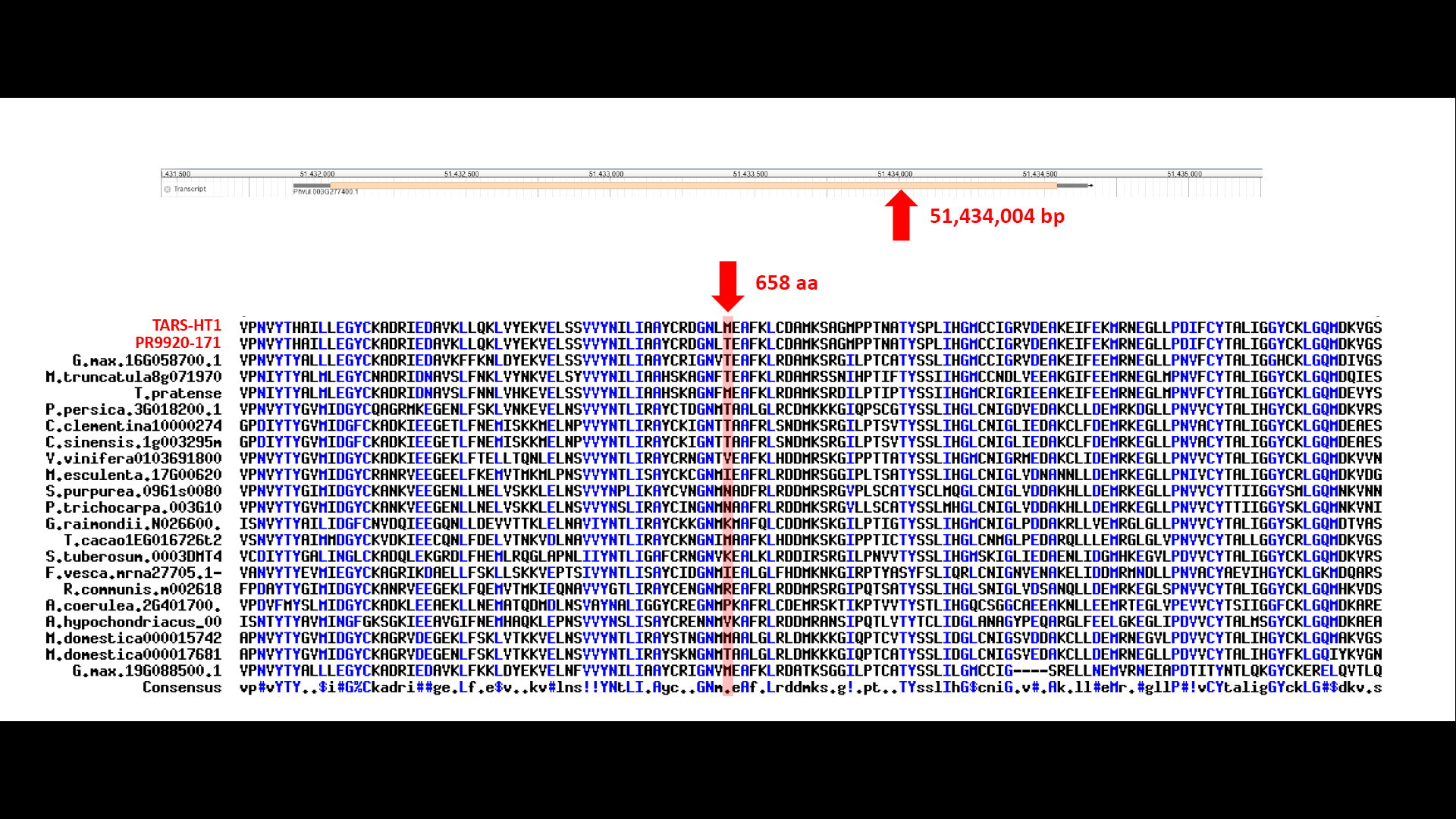


Figure S5. The conservation of the non-synonymous point mutation in the *Phvul.003G277400* coding region among homologous genes in other species. This mutation (highlighted) at position 658 aa converts a threonine (T) in PR9920-171 to a methionine (M) in TARS-HT1. This position is not located in a conserved domain.


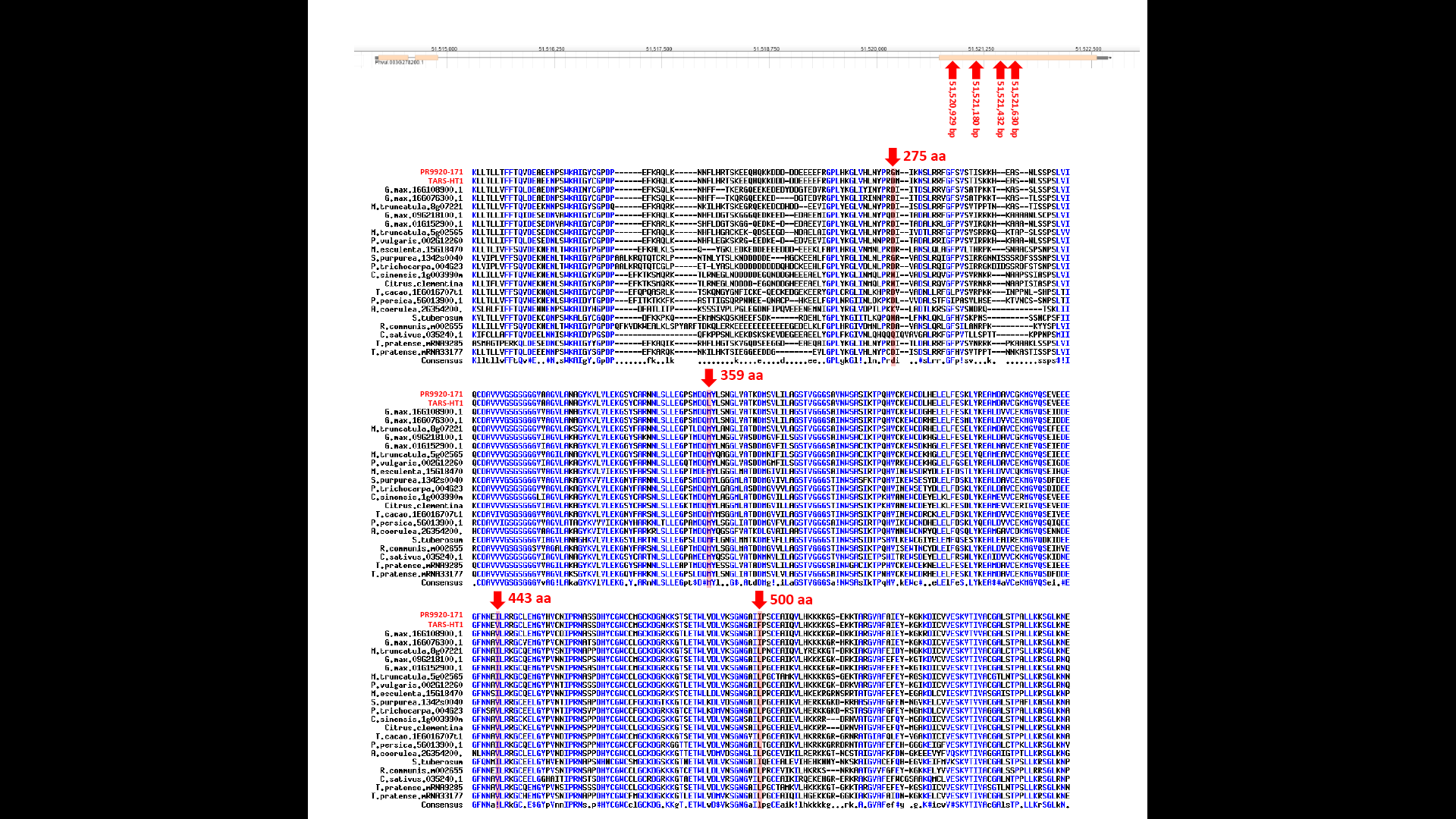


Figure S6. The conservation of the non-synonymous point mutations in the *Phvul.003G278200* coding region among homologous genes in other species. Four non-synonymous point mutations were detected at 275, 359, 443 and 500 aa. Among them, the mutations at 275 and 500 aa were located in a non-conserved site. The mutation at 443 was categorized as a partially conserved site, as both alleles in PR9920-171 (Isoleucine) and TARS-HT1 (Valine) were equally present among homologous genes. The mutation at 359 occurred at a conserved site that converted a Methionine in PR9920-171 to a Leucine in TARS-HT1. However, both aa are categorized as aa with hydrophobic side chains and thus likely don’t impose a drastic change in protein conformation.


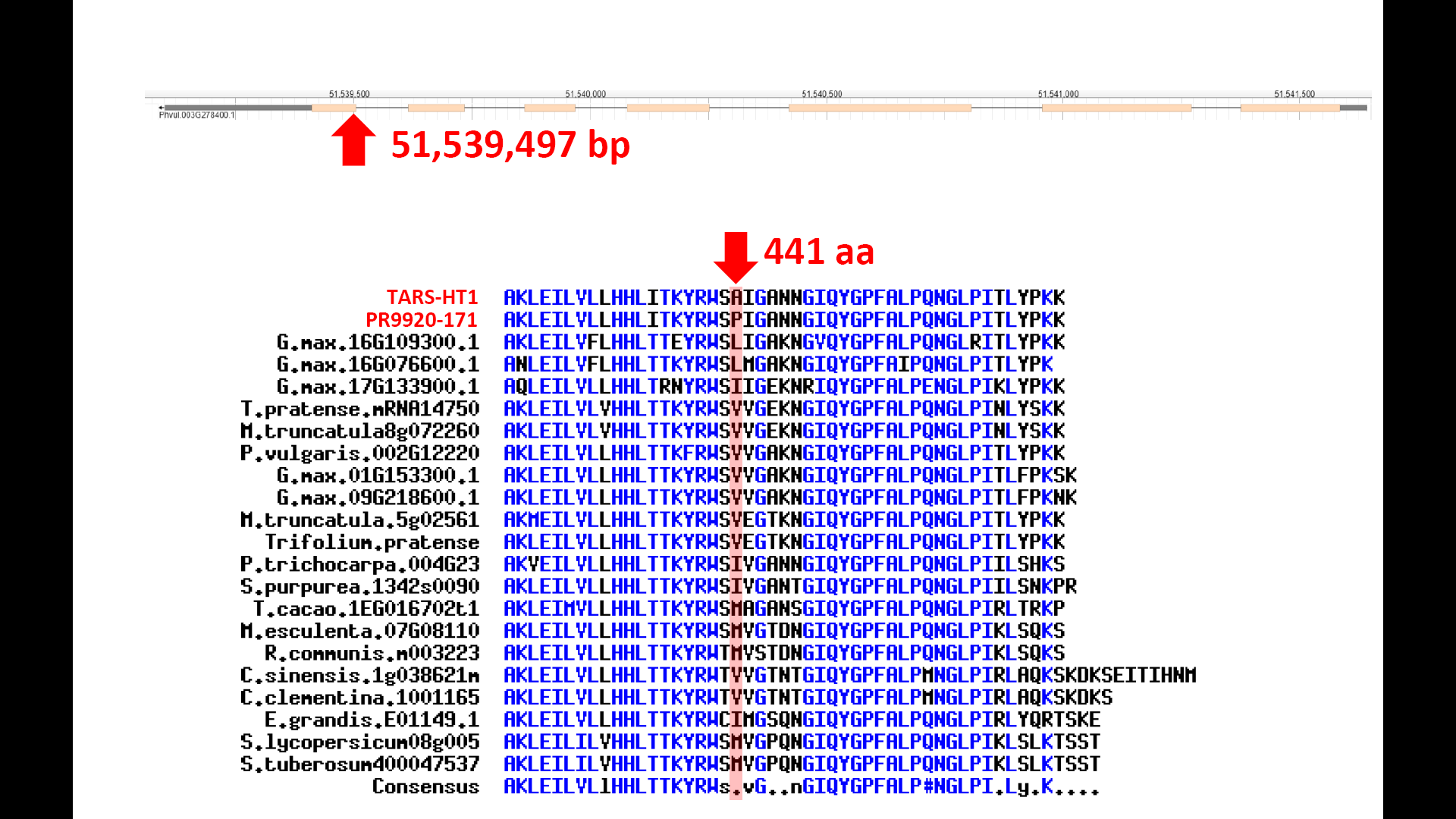


Figure S7. The non-synonymous point mutation in the *Phvul.003G278400* coding region at the last (7^th^) exon. This mutation (highlighted) at position 441 aa converts a Proline (P) in PR9920-171 to an Alanine (A) in TARS-HT1. This position is not located in a conserved domain.


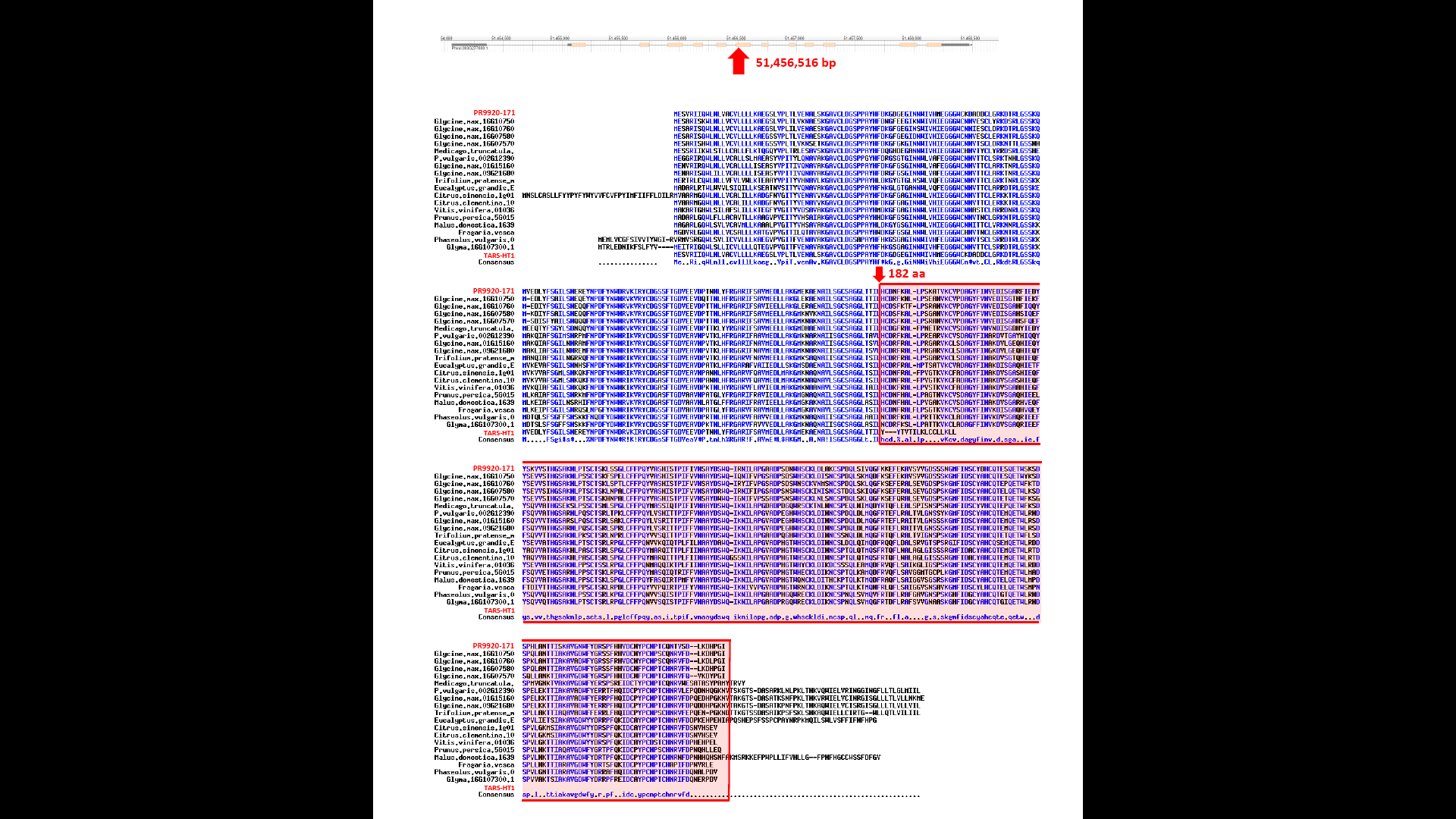


Figure S8. A 5-bp insertion in 6^th^ exon of *Phvul.003G277600* causes a frameshift in the aa sequence of TARS-HT1. This frameshift results in the aa change and truncation of almost half of the protein (red box) from 182 aa to the end at TARS-HT1. Alignment of this gene with homologous versions in other species reveals that this missing part contains several conserved domains. The TARS-HT1 sequence located at the bottom of alignment.
