# Supplementary material for: The genetics and physiology of seed dormancy, a crucial trait in common bean domestication
Source: BMC Plant Biol. 2021 Jan 22;21:58. doi: 10.1186/s12870-021-02837-6 (PMC7821524; doi:10.1186/s12870-021-02837-6)
Supplement: Supplementary file 2 — Additional file 2: Table S1. Field sites information [file 12870_2021_2837_MOESM2_ESM.docx]

| Table S1. The list of field sites used to evaluate trait (seed water uptake) stability | | | | |
| --- | --- | --- | --- | --- |
| Location | Abbreviation | Coordinates | Row per plot | Plot length (m) |
| Michigan | MI | 43°21'2.54"N 85°10'36.62"W | Single | 6 |
| Puerto Rico ^a^ | PR | 18°28'13.7"N 67°02'32.5"W | Single | 3 |
| Washington State | WA | 46°47'18.1"N 119°02'22.0"W | Double | 3 |
| ^a^ In PR, the TARS-HT1 plots had poor germination and did not produce seed | | | | |
